# Simultaneous and Visual Detection of KPC and NDM Carbapenemase-Encoding Genes Using Asymmetric PCR and Multiplex Lateral Flow Strip

**DOI:** 10.1155/2023/9975620

**Published:** 2023-07-22

**Authors:** Wei Lai, Yongjie Xu, Lin Liu, Huijun Cao, Bin Yang, Jie Luo, Ying Fei

**Affiliations:** ^1^School of Medical Laboratory, Guizhou Medical University, Guiyang 550004, Guizhou, China; ^2^NHC Key Laboratory of Pulmonary Immunological-Related Diseases, Guizhou Provincial People's Hospital, Guiyang 550002, Guizhou, China; ^3^The Center for Clinical Laboratories, The Affiliated Hospital of Guizhou Medical University, Guiyang 550004, China; ^4^Department of Laboratory Medicine, The Second People's Hospital of Guizhou Province, Guiyang 550002, China

## Abstract

Carbapenem-resistant *Enterobacteriaceae* (CRE) infections constitute a threat to public health, and KPC and NDM are the major carbapenemases of concern. Rapid diagnostic tests are highly desirable in point-of-care (POC) and emergency laboratories with limited resources. Here, we developed a multiplex lateral flow assay based on asymmetric PCR and barcode capture probes for the simultaneous detection of KPC-2 and NDM-1. Biotinylated barcode capture probes corresponding to the KPC-2 and NDM-1 genes were designed and cast onto two different sensing zones of a nitrocellulose membrane after reacting with streptavidin to prepare a multiplex lateral flow strip. Streptavidin-coated gold nanoparticles (SA-AuNPs) were used as signal reporters. In response to the target carbapenemase genes, biotin-labelled ssDNA libraries were produced by asymmetric PCR, which bond to SA-AuNPs via biotin and hybridise with the barcode capture probe via a complementary sequence, thereby bridging SA-AuNPs and the barcode capture probe to form visible red lines on the detection zones. The signal intensities were proportional to the number of resistance genes tested. The strip sensor showed detection limits of 0.03 pM for the KPC-2 and 0.07 pM for NDM-1 genes, respectively, and could accurately distinguish between KPC-2 and NDM-1 genes in CRE strains. For the genotyping of clinical isolates, our strip exhibited excellent consistency with real-time fluorescent quantitative PCR and gene sequencing. Given its simplicity, cost-effectiveness, and rapid analysis accomplished by the naked eye, the multiplex strip is promising auxiliary diagnostic tool for KPC-2 and NDM-1 producers in routine clinical laboratories.

## 1. Introduction

The number of infections caused by carbapenem-resistant *Enterobacteriaceae* (CRE) strains has increased in recent years [1, 2]. Carbapenemases are primarily classified into Ambler classes *A*, *B*, and *D* [3, 4]. Among CRE species, *Klebsiella pneumoniae* carbapenemase (KPC) and New Delhi-metallo-*β*-lactamase (NDM) pose a major threat, being the most prevalent in *Klebsiella pneumoniae* and *Escherichia coli*, respectively, in China [5] and causing an alarming increase in infection rates over the last year [6]. Infectious diseases caused by CRE have become an urgent concern for healthcare institutions because they are associated with high mortality and morbidity rates [7, 8]. Moreover, susceptible bacteria readily become resistant to carbapenems through the transfer of plasmids between *Enterobacteriaceae* [9, 10]. Thus, there is an urgent need for the rapid diagnosis of CRE infections.

Carbapenemase detection, which involves phenotypic screening and genotype detection, is the primary method used for the clinical diagnosis of CRE infections. Phenotypic tests include the Carba NP test [11], carbapenem inactivation method [12], modified Hodge test [13], carbapenemase inhibitor enhancement test [14], colour medium screening [15], and flight mass spectrometry [16]. These phenotypic screening methods show good specificity and low cost but often suffer from low sensitivity and slow turnaround times [17, 18]. Genotype determination mainly involves fluorescence quantitative PCR [19], enzyme immunochromatography technology [20–23], and genome sequencing [24]. Antibody-based immunochromatographic methods are fast and easy to operate but require expensive antibodies. Genotyping methods significantly increase the detection efficiency with high accuracy and sensitivity [25, 26]; however, the requirement for expensive instruments and strict experimental conditions limit their application in on-site detection and in remote areas.

Recently, lateral flow test (LFT) technology was applied in rapid nucleic acid detection [27–29]. Given its simplicity, rapid turnaround, low cost, and user-friendliness, LFT has become one of the most widely used point-of-care testing (POCT) methods for rapid diagnostic systems [30, 31]. Notably, LFT is further exploited for the simultaneous detection of multiple analytes from a single sample, commonly known as multiplexed detection, which has attracted considerable attention [21, 22, 32]. Multiplex testing reduces sample volume, analysis time, and cost. Owing to these advantages, LFT is widely used to detect drugs of abuse [33], nucleic acids [34], proteins [35], and bacteria [36–38].

In this study, we propose a multiplex assay for detecting KPC and NDM genes using an asymmetric PCR technique and a barcode-capture probe-based lateral flow test strip. An asymmetric PCR-based technique was employed to obtain adequate amounts of biotinylated single-stranded DNA products of KPC and NDM (biotin-ssDNA). Biotin-ssDNA reacts with streptavidin-coated gold nanoparticles (SA-AuNPs) on the conjugate pad to form a AuNP-SA-biotin-ssDNA (AuNP-ssDNA) complex. AuNP-ssDNA is hybridised to barcode capture probes when the sample flows through the test lines; thereby, AuNPs are immobilised on the test zones of the strip sensor to produce a red line. The visual signals could be read by the naked eye and quantified with a GIC-H1 portable analyser. The multiplex strip for typing KPC-2 and NDM-1 genes has great potential for application in basic microbiology laboratories.

## 2. Materials and Methods

### 2.1. Materials and Reagents

#### 2.1.1. Bacterial Strains

Bacterial strains were obtained from the Microbiology Laboratory of the Guizhou Provincial People's Hospital (Guiyang, China) and are listed in Table S1. The bacterial strains are provided with detailed information of the five main carbapenemases (KPC-, NDM-, VIM-, and IMP-type and OXA-48) in Figure S1.

#### 2.1.2. Construction of pUC57 Plasmids

The recombinant plasmids for KPC and NDM were named pUC57/kpc and pUC57/ndm (Figure S2), respectively, and the sequences of the DNA fragments are shown in Table S2 of Supplementary Material. For KPC-2 and NDM-1 gene testing, the two plasmids (pUC57/kpc and pUC57/ndm) were artificially synthesised and not isolated from bacteria, comprising partial KPC-2 and NDM-1 sequences (Tsingke Biotechnology Co., Ltd., Beijing, China). The synthesised plasmid did not contain any other beta-lactamase genes. The concentrations of the pUC57/kpc and pUC57/ndm plasmids were first determined by NanoDrop spectrophotometry.

#### 2.1.3. Reagents

The PCR Master Mix Assay kit and the DL500 DNA marker were purchased from Takara Bio Inc. (Dalian, China). GoldView-I nucleic acid stain, streptavidin, PEG-20000, and sodium chloride were obtained from Solarbio Science & Technology Co. Ltd. (Beijing, China). Tris-(hydroxymethyl) aminomethane (Tris), HAuCl_4_·3H_2_O, and Tween-20 were bought from Sangon Biotechnology Inc. (Shanghai, China). A MagIso DNA/RNA isolation kit was obtained from Xi'an Tianlong Science and Technology Co. Ltd. (Xian, China). A sample pad, a conjugate pad, absorbent paper, and a PVC baseboard were obtained from Jie Yi Biotechnology Co. Ltd. (Shanghai, China). UniSart CN 140 nitrocellulose membranes were obtained from Millipore (Billerica, MA, USA). Synthetic oligonucleotides were obtained from Tsingke Biotechnology Co. Ltd. (Beijing, China).

#### 2.1.4. Instruments

The instruments used mainly included VITEK® 2 Compact from BioMerieux (Mariere, France), MALDI Biotyper from Bruker corporation (Karlsruhe, Germany), automatic nucleic acid extraction instrument from Tianlong Technology (Xian, China), Biometra TAdvanced 96SG PCR from Analytik Jena (Jena, Germany), and HM3035 XYZ platform dispenser from Shanghai Kinbio Tech. Co. Ltd. (Shanghai, China) using a GIC-H1 portable colloidal gold analyser (Suzhou Hemai Precision Instrument Co., Ltd., Suzhou, China).

### 2.2. Methods

#### 2.2.1. Primer Design

KPC (NC_016846.1) and NDM (CP091986.1) genes were selected from GenBank (https://www.ncbi.nlm). Multiple pairs of primers were designed using Primer Premier 6.0 (Premier Biosoft, San Francisco, CA, USA) and Oligo 6 software (DBA Oligo, Inc., Colorado Springs, CO, USA); the forward and reverse primers for KPC-2 and NDM-1 are detailed in Table S2.

#### 2.2.2. Strain Culture and DNA Extraction

Bacterial strains were cultured on Columbia blood agar plates, and total DNA was extracted using the MagIso DNA/RNA isolation kit according to the manufacturer's instructions. The extracts were stored at −20°C before use.

#### 2.2.3. Establishment of the Asymmetric PCR System

Asymmetrical PCR of strains carrying the KPC-2 and NDM-1 genes yielded the corresponding biotin-labelled target single-stranded DNA (biotin-ssDNA). Under standard PCR conditions, different concentrations of forward and reverse primers were used. The asymmetrical PCR procedure was as follows: 95°C predenaturation for 3 min; 40 cycles, denaturation, annealing, and extension (95°C, 30 s; 55°C, 30 s; and 72°C, 20 s); and finally 72°C and extension for 5 min. PCR products were processed by electrophoresis at 120 V on 2.0% agar gel for 30 min, stained, and imaged using a gel imager. To obtain the best experimental conditions for asymmetric PCR, the annealing temperature gradient was optimized from 50 to 60°C. The concentration ratios of forward to reverse primers were 1 : 10, 1 : 20, 1 : 40, 1 : 60, 1 : 80, and 1 : 100. The number of asymmetric PCR cycles was 25, 30, 35, 40, 45, and 50. Finally, the reaction system (20 *μ*L volume) contained 10 *μ*L 2× PCR Master Mix (20 mM Tris-HCl, 100 mM KCl, 3 mM MgCl_2_, 500 *μ*M dNTP each, polymerase 0.1 U/*μ*L, and ddH_2_O) and 2 *μ*L template (plasmids or bacterial strain DNA extracts) diluted forward primers of KPC-2 and NDM-1 (0.6 *μ*M and 1 *μ*L for each gene), biotin-labelled reverse primers of KPC-2 and NDM-1 (20 *μ*M and 0.6 *μ*L for each gene), and 4.8 *μ*L ddH_2_O.

#### 2.2.4. Preparation and Modification of Gold Nanoparticles

All glassware used in the preparation was thoroughly cleaned in aqua regia (three parts HCl and one part HNO_3_), rinsed in double-distilled water, and oven-dried prior to use. Gold nanoparticles (AuNPs) were prepared using the citrate reduction method. Briefly, 100 mL of 0.01% HAuCl_4_ in double-distilled water was boiled and mixed with 4 mL 1% trisodium citrate with vigorous stirring. When the colour turned wine red, the heating source was withdrawn, and the colloid solution was stirred for another 15 min. The colloidal gold solution was photographed using TEM. Prior to modification, the pH of the AuNP solution was adjusted to 8.0 by addition of 0.1 mol/L K_2_CO_3_. Next, 1 mL AuNP solution was mixed with 20 *μ*L streptavidin solution (0.1 mg/mL). The mixture was shaken for 1 h, followed by addition of 0.1% BSA as a blocker. The mixture was incubated for 30 min at room temperature. The solution was centrifuged to remove unconjugated streptavidin at 10000 rpm for 20 min at 4°C. The pellet was reconstituted in 20 mL of the proper storage buffer (1% PBS, 1% BSA, 0.25% sucrose, 1% Tween-20, and 0.05% NaN_3_). The SA-AuNPs were finally stored at 4°C for further use. The absorption spectra of the AuNP and SA-AuNP solutions were scanned at *λ* 300–750 nm with a UV spectrophotometer.

#### 2.2.5. Preparation of Streptavidin-Biotin-DNA Probe Conjugates

The capture probes on test lines 1 and 2 (*T*1 and *T*2) were designed to immobilise the AuNP conjugates corresponding to KPC-2 and NDM-1, respectively. Three types of capture probe (Cp) were prepared: streptavidin-biotinylated Cp1 conjugates for *T*1 (KPC-2), streptavidin-biotinylated Cp2 conjugates for *T*2 (NDM-1), and streptavidin-biotinylated Cp3 conjugates for the control line. Streptavidin was dissolved in 10 mM (phosphate-buffered saline (PBS), pH 7.4) to a final concentration of 0.1 mg/mL. Next, 8 *μ*L streptavidin (0.1 mg/mL) and 1 *μ*L Cp (50 *μ*M) with a ratio of 8 : 1 were incubated at room temperature for 1 h to provide stable binding. The sample solutions were filtered (30 kDa, Amicon Ultra, Merck Millipore, Burlington, MA, USA) for 30 min at 6000 rpm to remove free Cp. Conjugated streptavidin-biotin-Cp, which remained in the filter owing to the large molecular size of streptavidin, was removed from the filter membrane and resuspended. Streptavidin-biotinylated-Cp conjugates were dispensed onto three regions of a nitrocellulose membrane to prepare two test lines and a control line.

#### 2.2.6. Detection of KPC and NDM Genes

DNA extracts from different strains, which contained with or without KPC and NDM gene, were subjected to the established asymmetric PCR system. Two types of plasmid DNA containing the KPC-2 or NDM-1 genes, used as standards, were tested. ddH_2_O was used as a blank control. Next, 10 *μ*L asymmetric PCR amplification products and 90 *μ*L 0.01 mol/L PBS diluent were mixed in an Eppendorf tube. Finally, the lateral flow strip was placed in the mixture for 15 min. The corresponding intensities were measured using the GIC-H1 portable analyser that scanned the test and control line and read the intensity of the reflected light.

#### 2.2.7. Carbapenemase Detection for Clinical Strains

To validate our method, we used 23 clinical isolates of *Enterobacteriaceae* to evaluate the KPC and NDM genes; these strains were either positive or negative for KPC or NDM. We used our strip and sequencing methods, and we compared the results between the methods. A meropenem disk with phenylboronic acid (PBA) and EDTA has also been used to detect double carbapenemase producers (serine and metallo-*β*-lactamase, respectively) [11, 13].

## 3. Results and Discussion

### 3.1. Principle of the Multiplex Strip for Sensing KPC and NDM Genes

**Figure 1:**
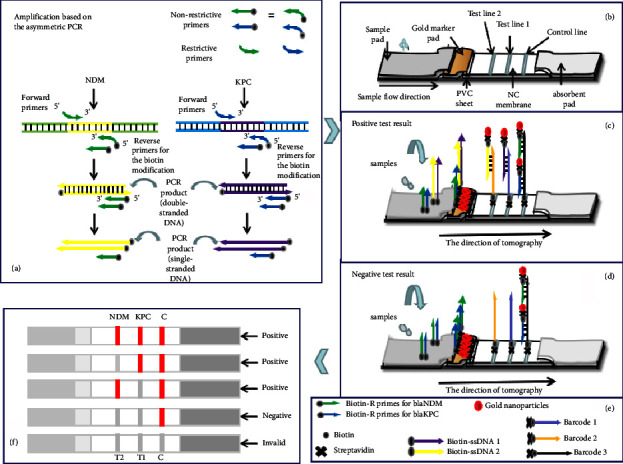
Principle of the multiplex strip for sensing KPC and NDM genes based on barcode capture probes and asymmetrical PCR. (a) Construction of the asymmetric PCR system for amplifying KPC-2 and NDM-1. (b) Design and configuration of the barcode colloid gold strip based on asymmetric PCR. (c) Binding and colour development signal of KPC and NDM genes (positive test). (d) Binding and colour development signal in the absence of KPC and NDM genes (negative test). (e) Interpretation of reagents and probes used in the strip and asymmetrical PCR system. (f) Appearance of the control line demonstrated valid test results; appearance of one or two test lines indicated a positive result for the corresponding carbapenemase encoding genes; no test line indicated a negative result for the two carbapenemase gene; the disappearance of the control line demonstrated invalid test results.

The working principle of the nucleic acid lateral-flow test strip is shown in Figure 1. In this study, an integrated multiplex assay based on asymmetric PCR techniques and barcode lateral flow tests was used to detect genes in CRE strains. First, primers for KPC-2 and NDM-1 genes were designed with reference to the GenBank data on KPC and NDM genes. To react with streptavidin-coated AuNPs, the reverse primers of the KPC-2 and NDM-1 genes were tailored with a biotin molecule at their 5′ end. Upon amplification of the target carbapenem-resistant gene by asymmetric PCR, a library of asymmetric PCR products (biotin-ssDNA) was produced. Biotin-ssDNA1 was generated in the presence of KPC-2, and biotin-ssDNA2 corresponded to the NDM-1 gene. With co-occurrence of KPC-2 and NDM-1, the asymmetric PCR products consisted of biotin-ssDNA1 and biotin-ssDNA2 (Figure 1(a)). Three types of biotin-DNA barcode Cps were designed: biotin-Cp1 complementary to the partial sequence of biotin-ssDNA1 (KPC-2), biotin-Cp2 complementary to the partial sequence of biotin-ssDNA2 (NDM-1), and biotin-Cp3 complementary to the reverse primer sequences of KPC-2 and NDM-1. These biotin-capture probes reacted with streptavidin to form a streptavidin-biotin-Cp complex (barcodes 1, 2, and 3), which was dispensed onto different zones of the nitrocellulose membrane as capture probes for the test and control lines; *T*1 and *T*2 corresponded to KPC-2 and NDM-1, respectively. AuNP-SA conjugates were employed as reporters and loaded onto the conjugate pad. Visual detection of the sample reaction solution was performed using a strip-sensing system consisting of four components: a sample pad, a conjugate pad, a nitrocellulose membrane, and an absorption pad (Figure 1(b)). The presence of KPC-2 and/or NDM-1 resulted in the generation of biotin-ssDNA products. A sample solution containing the biotin-ssDNA products was then applied to the sample pad. The solution was migrated by capillary action, passed through the conjugate pad, and rehydrated the AuNP-SA conjugates. Biotin-ssDNA reacts with AuNP-SA to form AuNP-SA-biotin-ssDNA complexes (AuNPs-SA-biotin-ssDNA) which continue to migrate along the strip. Because the ssDNA products were complementary to the barcode on the immobilised test lines, the above complexes were captured in the test zones by hybridisation between ssDNA and barcode probes. The accumulation of AuNPs in test zones 1 and 2 is visualised as characteristic red bands. Excess biotin-labelled reverse primers reacted with AuNPs-SA conjugates to form AuNPs-SA-biotin-R primers, which continued to migrate and were captured in the control zone by hybridisation with barcode 3, thus generating a third red band (Figure 1(c)). In the absence of KPC-2 and NDM-1, no red band was observed in the test zone owing to the failure of ssDNA production. The biotin-modified reverse primers of the KPC-2 and NDM-1 genes were excess, and a red band was always formed in the control zone, confirming the proper functioning of the strip biosensor (Figure 1(d)). Figure 1(e) shows the interpretation of reagents and probes used in the strip and asymmetrical PCR system. The positive results of KPC-2, NDM-1, both KPC-2 and NDM-1, and invalid test result are explained in Figure 1(f).

### 3.2. Characterization of the Prepared and Modified Gold Nanoparticles

**Figure 2:**
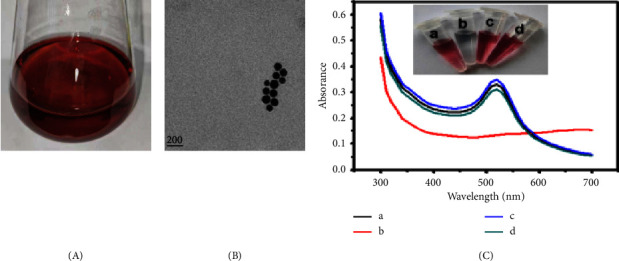
Characterization of the prepared and modified colloidal gold. (A) Colloidal gold solution. (B) TEM image of gold nanoparticles. (C) Colour observation and UV spectra analysis of gold nanoparticle solution without (a) and with (b) a high salt concentration; streptavidin-modified gold nanoparticles without (c) and with (d) a high salt concentration.

The colloidal gold solution prepared by citrate reduction was clear red (Figure 2(a)). The gold nanoparticles were dispersed with a diameter of 30–50 nM, as confirmed by transmission electron microscopy (TEM, Figure 2(b)). A typical absorption peak at *λ* 520 nm was observed in the colloidal gold solution using the UV spectrophotometer (tube *a* and spectrum *a*, Figure 2(c)). These findings indicated that colloidal gold was successfully prepared. After modification, the streptavidin-coated colloidal gold solution remained red (tube *c*), and the corresponding absorption peak at 520 nm remained unchanged (spectra *c*). Upon addition of 10% sodium chloride to the colloidal gold solution, the colour changed from burgundy to grey (tube *b*). However, adding sodium chloride had an effect on the colour change (tube *d*), and absorbance peaks at *λ* 520 nm were detected by using the UV spectrophotometer. This occurred because outer streptavidin prevented the aggregation of colloidal gold in a high-salt solution, suggesting successful modification of colloidal gold with streptavidin.

### 3.3. Feasibility of the Multiplex Strip for Sensing KPC-2 and NDM-1

**Figure 3:**
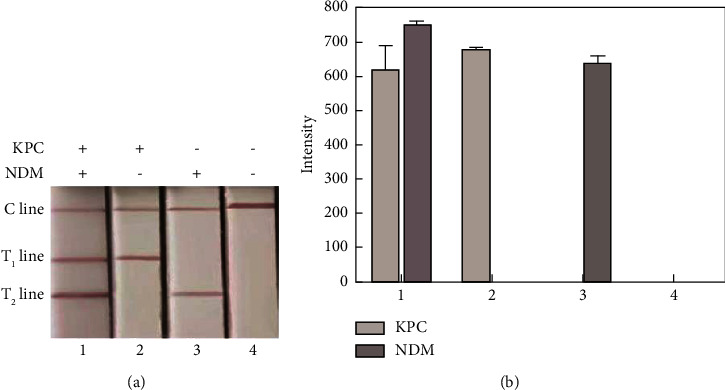
Feasibility of the multiplex strip for sensing KPC and NDM genes. (a) Strips in response to different assay systems containing pUC57/kpc and pUC57/ndm (1), pUC57/kpc (2), pUC57/ndm (3), and blank (4). (b) Corresponding intensities of the multiplex strips. Error bars represent the standard deviation of three independent measurements.

We investigated the feasibility of our method by using two types of plasmids containing KPC-2 (pUC57/kpc) and NDM-1 (pUC57/ndm) sequences (100 pM) as targets. The presence of both the KPC-2 and NDM-1 genes resulted in two red test lines (Figure 3), indicating that the biotin-ssDNA libraries successfully connected AuNPs-SA onto the barcode probes of KPC-2 and NDM-1. The presence of KPC-2 or NDM-1 led to a single red test line in the test zone. These results suggest that only one type of biotin-ssDNA was generated in response to KPC-2 or NDM-1, which immobilised AuNPs-SA on the test line. Only the control line appeared in the absence of KPC-2 and NDM-1. Therefore, the established method has a robust ability to accurately and rapidly detect carbapenemase genotypes.

### 3.4. Optimization of the Asymmetric PCR Reaction System

#### 3.4.1. Optimization of the Asymmetric PCR Annealing Temperature

**Figure 4:**
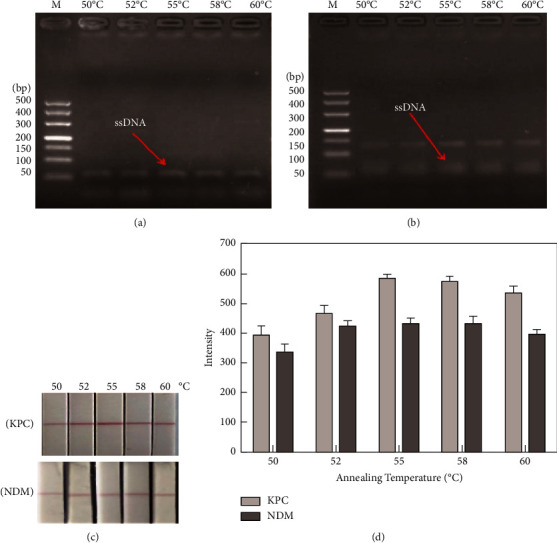
Optimization of annealing temperature for asymmetric PCR. (a) Asymmetric PCR biotin-ssDNA products for KPC-2 and (b) NDM-1 at varied annealing temperatures by agarose gel electrophoresis. (c) Visual signal of the lateral strip for KPC-2 and NDM-1 at different annealing temperatures and (d) the corresponding intensity detected by using the GIC-H1 portable analyser.

Annealing temperature is an important experimental parameter affecting the analytical performance of asymmetric PCR. The maximum production of the target biotin-ssDNA and minimum production of by-products were achieved at 55°C for the KPC-2 gene at 10 pM (Figure 4(a)). Meanwhile, the test line of KPC-2 showed the highest signal (585.33, Figures 4(c) and 4(d)). For the NDM-1 gene at 10 pM, the amount of biotin-ssDNA product for NDM-1 at 55°C was marginally higher than that at other temperatures (Figure 4(b)). With the biotin-ssDNA products on the strip, a relatively stronger signal of 435.33  was obtained at 55°C (Figures 4(c) and 4(d)). Therefore, 55°C was selected as the best annealing temperature for amplifying KPC-2 and NDM-1.

#### 3.4.2. Optimization of the Ratio of the Forward to Reverse Primer

**Figure 5:**
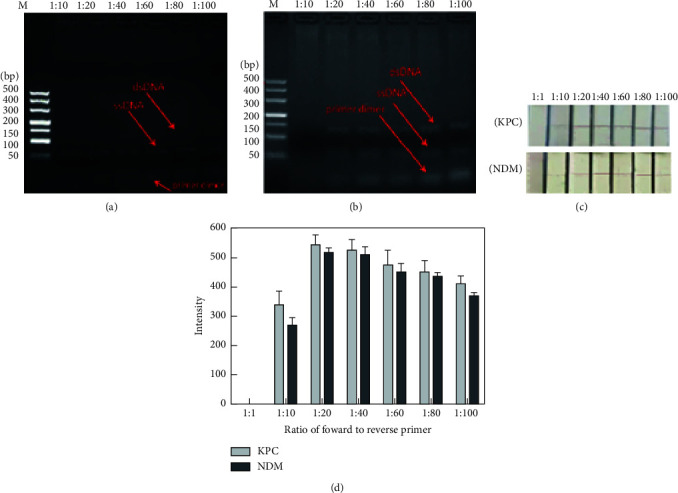
Optimization of the ratio of forward to reverse primer for asymmetric PCR. (a) Biotin-ssDNA products for KPC-2 and (b) NDM-1 at varied *F*/*R* ratios analysed by gel electrophoresis. (c) Visual signal of the lateral strip for KPC-2 and NDM-1 at varied *F*/*R* ratios and (d) the corresponding intensity detected by using the GIC-H1 portable analyser.

The ratio of forward to reverse primers (F/R ratio) is an important factor of biotin-ssDNA production. In the preparation of target biotin-ssDNA, the limited forward primers would be exhausted and the excess reverse primers would amplify the dsDNA in the early phase of asymmetric PCR to produce numerous ssDNA amplicons. At 10 pM of the target gene of KPC-2 and NDM-1, we observed a small difference between the gel electrophoresis bands of asymmetric PCR at different primer ratios (Figures 5(a) and 5(b)). However, the strip signals in Figure 5(c) appear different in staining, and the corresponding signal intensity in Figure 5(d) shows that the 1 : 20 *F*/*R* ratio was optimized based on the high signal intensity of KPC-2 and NDM-1 (544.67  and 515.33, respectively). Therefore, an *F*/*R* ratio of 1 : 20 was selected for subsequent assays.

#### 3.4.3. Optimization of the Cycle Number

**Figure 6:**
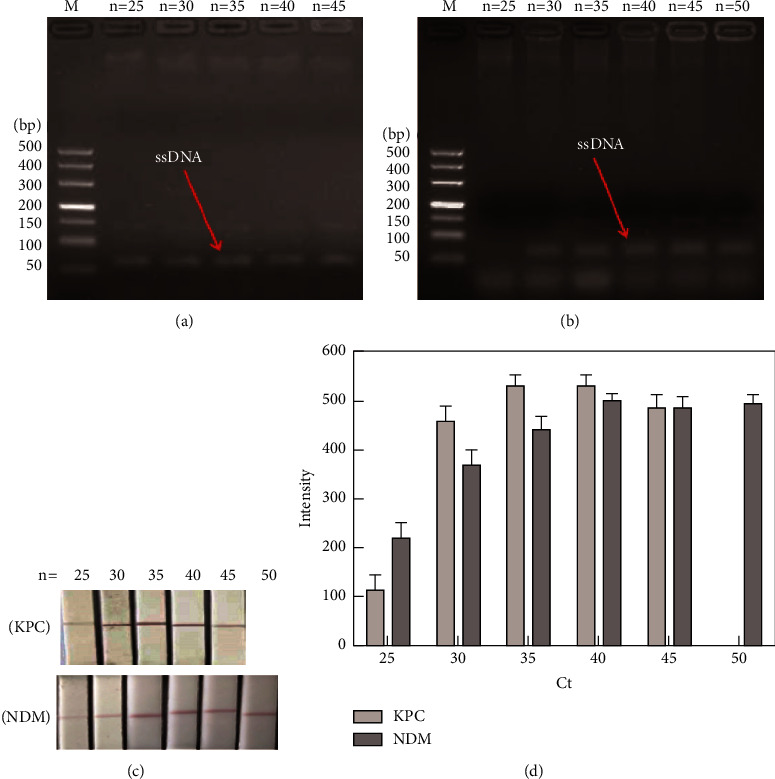
Optimization of the cycle number for asymmetric PCR. (a) Biotin-ssDNA products for KPC-2 and (b) NDM-1 at varied cycle numbers analysed by agarose gel electrophoresis. (c) Visual signal of the lateral strip for KPC-2 and NDM-1 at varied cycle numbers and (d) the corresponding intensity detected by using the GIC-H1 portable analyser.

In asymmetric PCR, the cycle number is a vital factor affecting the production of biotin-ssDNA and thus needed to be optimized. With an increase in the cycle number, the production of biotin-ssDNA for KPC-2 (10 pM) increased and tended to peak at 35 cycles (Figure 6(a)). The strip achieved its highest intensity (533.33 ) after 35 cycles (Figures 6(c) and 6(d)). Agarose gel electrophoresis of the DNA bands showed that biotin-ssDNA products for NDM-1 (10 pM) increased as the number of cycles increased from 25 to 40 and decreased thereafter (Figure 6(b)). For the strip, the maximum signal (505.33 ) for NDM-1 occurred at 40 cycles (Figures 6(c) and 6(d)), which is consistent with the agarose gel electrophoresis analysis. Considering the compatibility of the asymmetric PCR system for KPC-2 and NDM-1, the best assay conditions were selected at 40 cycles.

### 3.5. Sensitivity Analysis

**Figure 7:**
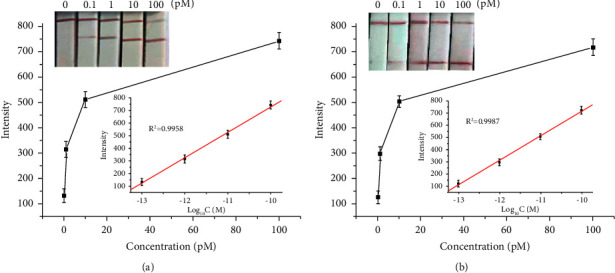
Sensitivity investigation. (a) Detection results of the strip at different concentrations of KPC-2 and the corresponding linear relationship of signal intensity and logarithm of the concentration of KPC-2. (b) Detection results of the strip at different concentrations of NDM-1 and the corresponding linear relationship of signal intensity and logarithm of the concentration of NDM-1.

To investigate the sensitivity of the strip sensor and establish standard curves for KPC-2 and NDM-1, the concentrations of the pUC57/kpc and pUC57/ndm plasmids were set in 10-fold dilution. Under optimized experimental conditions, pUC57/kpc and pUC57/ndm were added to the asymmetric PCR system, and the obtained biotin-ssDNA products were applied to the test strip. The strip signals were enhanced with increasing concentrations of the target KPC-2 and NDM-1, and the strip intensities were proportional to the logarithm of the KPC-2 and NDM-1 concentrations (from 0.1 to 100 pM, Figures 7(a) and 7(b)). The resulting calibration curves were *I* = 2746.32 + 201.82log_10_C for KPC-2 and *I* = 2700.61 + 199.03log_10_C for NDM-1 (inset in Figures 7(a) and 7(b)). According to the principle of blank plus three times of standard, the detection limits were down to 0.03 pM for KPC-2 and 0.07 pM for NDM-1. The red bands on the test zone were clearly visible even at 0.1 pM of KPC-2 and NDM-1, suggesting that the multiplex strip can be used for simultaneous visual detection of KPC and NDM genes without any specialized instrumentation. Therefore, this multiplex strip shows great promise for POCT detection of KPC-2 and NDM-1—the main markers of CRE.

### 3.6. Specificity Analysis

**Figure 8:**
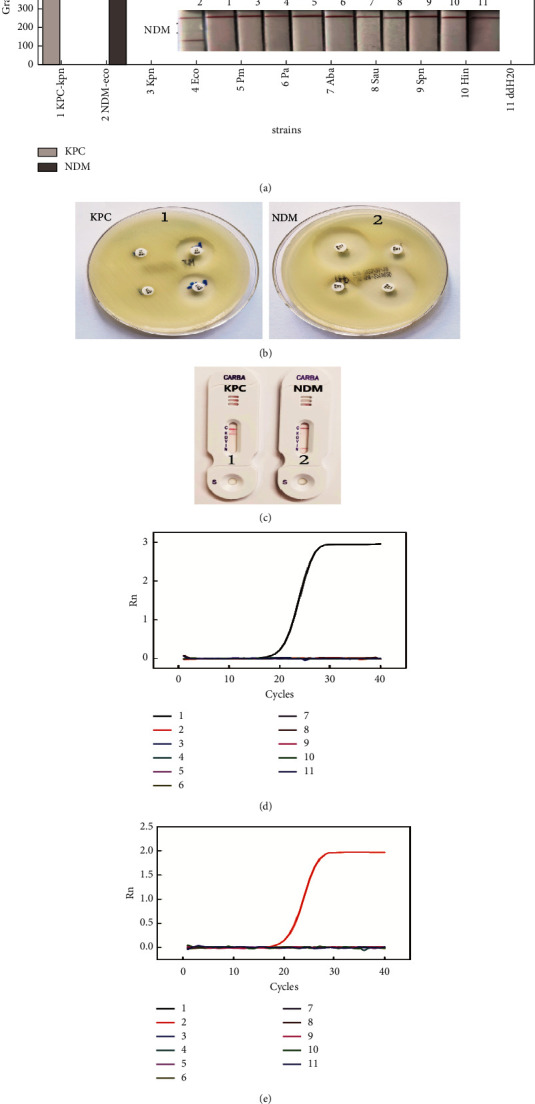
Specificity investigation. (a) Genotyping of KPC and NDM using the multiplex strip. (b) Phenotyping of KPC and NDM enzymes based on drug susceptibility. (c) Detection of KPC and NDM enzymes using the antibody-based lateral flow immunochromatographic strip. (d) Detection of the KPC gene using real-time fluorescence PCR. (e) Detection of the NDM gene using real-time fluorescence PCR. Error bars represent the standard deviation of three independent measurements.

Specificity of the strip sensor was evaluated by adding a set of bacterial strains to the asymmetric PCR system. Two clinical strains carrying carbapenemase and eight standard strains carrying no carbapenemase were selected to test assay specificity (Table 1). The asymmetric PCR products were sampled on lateral flow strips, and the results were observed after 10 min. The results indicated the presence of KPC-2 in carbapenem-resistant* K. pneumoniae* and NDM-1 in carbapenem-resistant *E. coli*, while the eight standard strains including *K. pneumoniae* and *E. coli* showed negative results, similar to those for the blank control (ddH_2_O, Figure 8(a)). Fluorescence PCR, a sensitive method for the detection of carbapenemase genes [39], was performed on the aforementioned strains , which showed that the genotyping of the KPC and NDM genes was 100% consistent with the multiplex strip (Figures 8(d) and 8(e)). In addition, the phenotypes of the KPC and NDM enzymes were analysed by assessing the drug susceptibility of two carbapenem-resistant strains isolated from clinical samples. Imipenem-resistant *K. pneumoniae* and *Escherichia coli* became sensitive after the addition of boric acid and EDTA solution, respectively (Figure 8(b)). The changes in susceptibility suggest that *K. pneumonia*e produced the KPC enzyme, the activity of which was inhibited by boric acid, and *E. coli* produced the NDM enzyme, the activity of which was blocked by EDTA. The lateral flow test for typing carbapenemases further confirmed the existence of the KPC enzyme in *K. pneumoniae* and the NDM enzyme in *E. coli* (Figure 8(c)). For the two clinical CRE strains, the phenotypes of the KPC and NDM enzymes were consistent with the genotype test using fluorescent PCR and our strip test. In summary, this multiplex strip could accurately detect CRE strains containing KPC and NDM genes and had no nonspecific reaction with other standard strains used in the assay.

### 3.7. Validation of the Strip for Testing KPC and NDM Genes in Clinical Samples

Of the 23 clinical samples tested, we detected 12 *K. pneumoniae* with KPC-positive genes, 1 *E. cloacae* with KPC-positive genes, 2 *K. pneumoniae* with NDM-positive genes, 2 *E. coli* with NDM-positive genes, 1 *E. oxytoca* with NDM-positive genes, and 5 *Enterobacter* without carbapenemase-resistant genes (Table 1). The identification of carbapenemase genes using the multiplex strip (Figure S3) was consistent with the sequencing results (Tables S3 and S4). We performed phenotypic tests of bacterial antimicrobial resistance and found consistent results with those of the strip and sequencing analysis. In tests with a small number of clinical samples, no false-positive or false-negative results were observed. The strip correctly identified all 18 isolates as carbapenemase-positive (KPC and NDM, sensitivity: 100%). The sensing probe used in the assay was also not cross-reactive (specificity: 100%). The results confirm that the strip sensor shows great potential in the typing of CRE genes in clinical samples.

## 4. Conclusion

The multiplex lateral flow strip was developed for the simultaneous and visual detection of KPC and NDM genes in CRE strains using asymmetric PCR, barcoded capture probes, and streptavidin-coated gold nanoparticles. Asymmetric PCR enabled the production of numerous biotin-ssDNAs with high sensitivity. DNA barcodes designed as capture probes on the test and control lines enabled multiplex detection of carbapenemase genes in a one-pot assay system. This method has a shorter assay time and higher specificity than traditional antimicrobial susceptibility testing methods. Compared with other available lateral flow immunoassays [40], our multiplex strip successfully detected the two major carbapenemases in CRE strains. Nucleic acid probe-based methods have the advantages of easy preparation, higher sensitivity owing to the integration of the amplification strategy, and lower costs than antibody-dependent lateral flow immunochromatographic assays [20–22]. Another advantage of this method is that the test strips can be stored at room temperature for longer periods [3]. Although the strip is convenient and user-friendly, it is limited by its reliance on ordinary PCR instruments and capacity for only detecting KPC-2 and NDM-1. Further work is required to adopt the isothermal amplification method and design a set of capture probes for sensing a greater variety of carbapenemase genes, including KPC, IMP, VIM, NDM, and OXA-48. Overall, the developed lateral flow assay exhibits high sensitivity and specificity, providing a promising method for POCT in limited-resource settings, and ultimately prevents CRE transmission and outbreaks in hospitals.

## Figures and Tables

**Table 1:** Evaluation of KPC and NDM detection in 23 clinical samples of *Enterobacteriaceae*.

No.	Bacterial isolates	Carbapenemase inhibitor enhancement test (phenotypic tests)	LFT results		Sequencing	
			KPC	NDM	KPC	NDM
1	*E. coli*	Metallo-ß-lactamase	*N*	*P*	*N*	NDM-1
2	*E. asburiae*	Noncarbapenemase producers	*N*	*N*	*N*	*N*
3	*K. pneumoniae*	Serine	*P*	*N*	KPC-2	*N*
4	*K. pneumoniae*	Serine	*P*	*N*	KPC-2	*N*
5	*K. pneumoniae*	Serine	*P*	*N*	KPC-2	*N*
6	*K. pneumoniae*	Serine	*P*	*N*	KPC-2	*N*
7	*K. pneumoniae*	Serine	*P*	*N*	KPC-2	*N*
8	*K. pneumoniae*	Serine	*P*	*N*	KPC-2	*N*
9	*K. pneumoniae*	Noncarbapenemase producers	*N*	*N*	*N*	*N*
10	*K. pneumoniae*	Serine	*P*	*N*	KPC-2	*N*
11	*E. cloacae*	Serine	*P*	*N*	KPC-2	*N*
12	*E. coli*	Metallo-ß-lactamase	*N*	*P*	*N*	NDM-1
13	*K. oxytoca*	Metallo-ß-lactamase	*N*	*P*	*N*	NDM-1
14	*K. pneumoniae*	Serine	*P*	*N*	KPC-2	*N*
15	*K. pneumoniae*	Serine	*P*	*N*	KPC-2	*N*
16	*E. coli*	Noncarbapenemase producers	*N*	*N*	*N*	*N*
17	*K. pneumoniae*	Serine	*P*	*N*	KPC-2	*N*
18	*E. coli*	Noncarbapenemase producers	*N*	*N*	*N*	*N*
19	*K. pneumoniae*	Metallo-ß-lactamase	*N*	*P*	*N*	NDM-1
20	*K. pneumoniae*	Serine	*P*	*N*	KPC-2	*N*
21	*K. pneumoniae*	Metallo-ß-lactamase	*N*	*P*	*N*	NDM-1
22	*K. pneumoniae*	Noncarbapenemase producers	*N*	*N*	*N*	*N*
23	*K. pneumoniae*	Serine	*P*	*N*	KPC-2	*N*

## Data Availability

The data used to support the findings of this study are available in the supplementary material of this article.

## Supplementary Materials

Supplementary Materials Concise description for each supplementary Figure and Table. Table S1 included control strains and clinical isolates used in this study. Table S2 included sequences of amplification primers and capture probes used in this study. Table S3 included the sequencing results for clinical strains carrying KPC-2 gene. Table S4 included the sequencing results for clinical strains carrying NDM-1 gene. Figure S1 included the test results of control strains and clinical isolates listed in Table S1 using Carba-5 strip. Figure S2 included information of plasmid pUC57 carrying blaKPC gene or blaNDM gene. Figure S3 included results of KPC-2 and NDM-1 testing on 23 *Enterobacteriaceae* samples using our strip.
