# Supplementary material for: Simultaneous and Visual Detection of KPC and NDM Carbapenemase-Encoding Genes Using Asymmetric PCR and Multiplex Lateral Flow Strip
Source: J Anal Methods Chem. 2023 Jul 22;2023:9975620. doi: 10.1155/2023/9975620 (PMC10386901; doi:10.1155/2023/9975620)
Supplement: Supplementary Materials — Concise description for each supplementary Figure and Table. Table S1 included control strains and clinical isolates used in this study. Table S2 included sequences of amplification primers and capture probes used in this study. Table S3 included the sequencing results for clinical strains carrying KPC-2 gene. Table S4 included the sequencing results for clinical strains carrying NDM-1 gene. Figure S1 included the test results of control strains and clinical isolates listed in Table S1 using Carba-5 strip. Figure S2 included information of plasmid pUC57 carrying blaKPC gene or blaNDM gene. Figure S3 included results of KPC-2 and NDM-1 testing on 23 Enterobacteriaceae samples using our strip. [file 9975620.f1.doc]

**Supplementary Information**

**Simultaneous and visual detection of KPC and NDM carbapenemase-encoding genes using asymmetric PCR and multiplex lateral flow strip**

Wei Lai^a^,[[1]](#footnote-2), Yongjie Xu^b,1^, Lin Liu^b^, Huijun Cao^a, d^, Bin Yang^b^, Jie Luo^c^, *, Ying Fei^a, d^, *

^a^ School of Medical Laboratory, Guizhou Medical University, Guiyang 550004, Guizhou, China

^b^ NHC Key Laboratory of Pulmonary Immunological--related Diseases, Guizhou Provincial People's Hospital, Guiyang 550002, Guizhou, China

^c^ Department of Laboratory Medicine, The Second People’s Hospital of Guizhou Province, Guiyang 550002, China

^d^ The Center for Clinical Laboratories, The Affiliated Hospital of Guizhou Medical University, Guiyang 550004, China

**Keywords**：Carbapenemase, KPC, NDM, Asymmetric polymerase chain reaction, Carbapenem-resistant *Enterobacteriaceae*, Lateral flow test, Gold nanoparticle, Nucleic acid detection

**Table S1** Control strains and clinical isolates used in this study.

| Number | Control strain | Abbreviation | Carbapenemase  in-house | Strain informations |
| --- | --- | --- | --- | --- |
| 1 | (KPC) Klebsiella pneumoniae | KPC-kpn | KPC | Clinical isolates |
| 2 | (NDM) *Escherichia. coli* | NDM-eco | NDM | Clinical isolates |
| 3 | *Klebsiella pneumoniae* | Kpn | Negative | CMCC46117 |
| 4 | *Escherichia. coli* | Eco | Negative | ATCC25922 |
| 5 | *Proteus mirabilis* | Pm | Negative | CMCC49005 |
| 6 | *Pseudomonas aeruginosa* | Pa | Negative | ATCC27853 |
| 7 | *Acinetobacter baumannii* | Aba | Negative | ATCC19606 |
| 8 | *Staphylococcus aureus* | Sau | Negative | ATCC25923 |
| 9 | *Streptococcus pneumoniae* | Spn | Negative | ATCC49619 |
| 10 | *Hemophilus influenzae* | Hin | Negative | ATCC49247 |

Note: The antibiotic susceptibility testing for all strains were performed. Strains 1 and 2 were resistant to meropenem and imipenem, and strains 3-10 were sensitive to meropenem and imipenem.


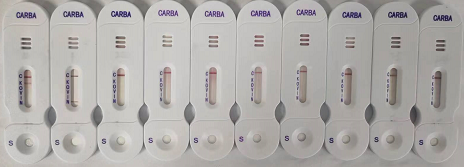


1 2 3 4 5 6 7 8 9 10

**Figure S1.** Control strains and clinical isolates listed in Table S1 were tested using Carba-5 strip.

Note: A lateral flow immunoassay (Carba-5) for the detection of the five main carbapenemases (KPC-, NDM-, VIM- and IMP-type and OXA-48).

**Table S2** Sequences of amplification primers and capture probes used in this study.

| Target Gene | Sequence (5′–3′) | 5′Modification |
| --- | --- | --- |
|  |  |  |
| *bla*KPC | F1: ttttttGGCTAAAGGGAAACACGACC |  |
|  | R1: ttttttCATAGTCATTTGCCGTGCCA | Biotin |
|  | Cp1: tttttttCGCATCCGCGCGGCGGTGCCGGCAGACTGGGCAGTC | Biotin |
| *bla*NDM | F2: ttttttATTGGCATAAGTCGCAATCC |  |
|  | R2: ttttttTCAACTGGATCAAGCAGGAG | Biotin |
|  | Cp2: tttttttCCGCCCATCTTGTCCTGATGCGCGTGAGTCACCACC | Biotin |
| Control | Cp3: tttttCTCCTGCTTGATCCAGAttttt*GGCACGGCAAATGACTATG* | Biotin |
| Puc57/kpc | ATT**GGCTAAAGGGAAACACGACC**GGCAACCACCGCATCCGCGCGGCGGTGCCGGCAGAC**TGGG**CAGTCGGAGACAAAACCGGAACCTGCGGAGTGTATGGCACGGCAAATGACTATGCC |  |
| Puc57/ndm | CATTGGCATAAGTCGCAATCCCCGCCGCATGCAGCGCGTCCATACCGCCCATCTTGTCCTGATGCGCGTGAGTCACCACCGCCAGCGCGACCGGCAGGTTGATCTCCTGCTTGATCCAGTTGA |  |

Note: F, forward primer; R, reverse primer; Cp, capture probe. The complementary region of Cp1 on Puc57/kpc is marked with red, while the complementary region of Cp2 on Puc57/ndm is marked with blue.


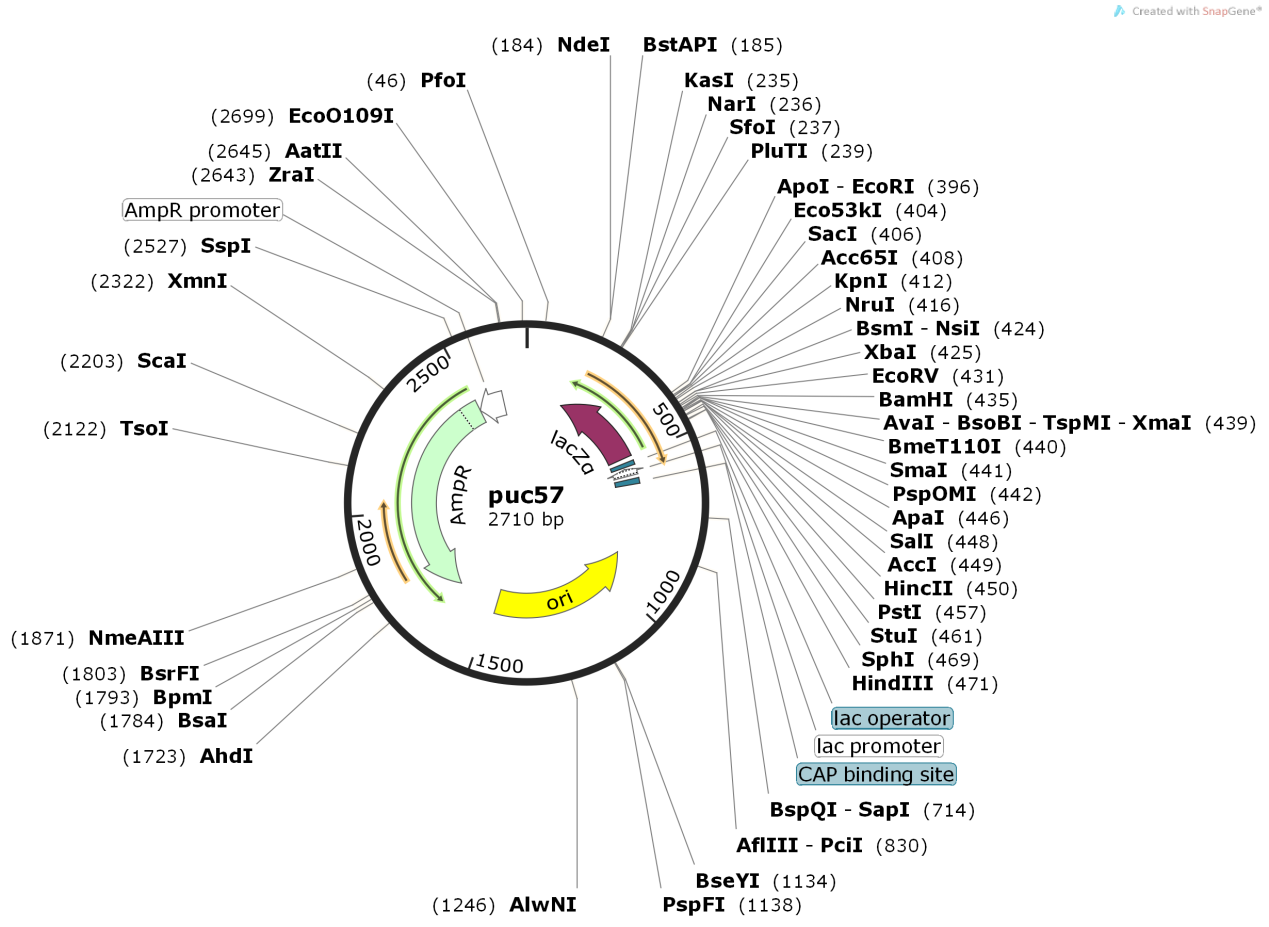


**Figure S2. The information of plasmid pUC57 carrying blaKPC gene or blaNDM gene.**

**
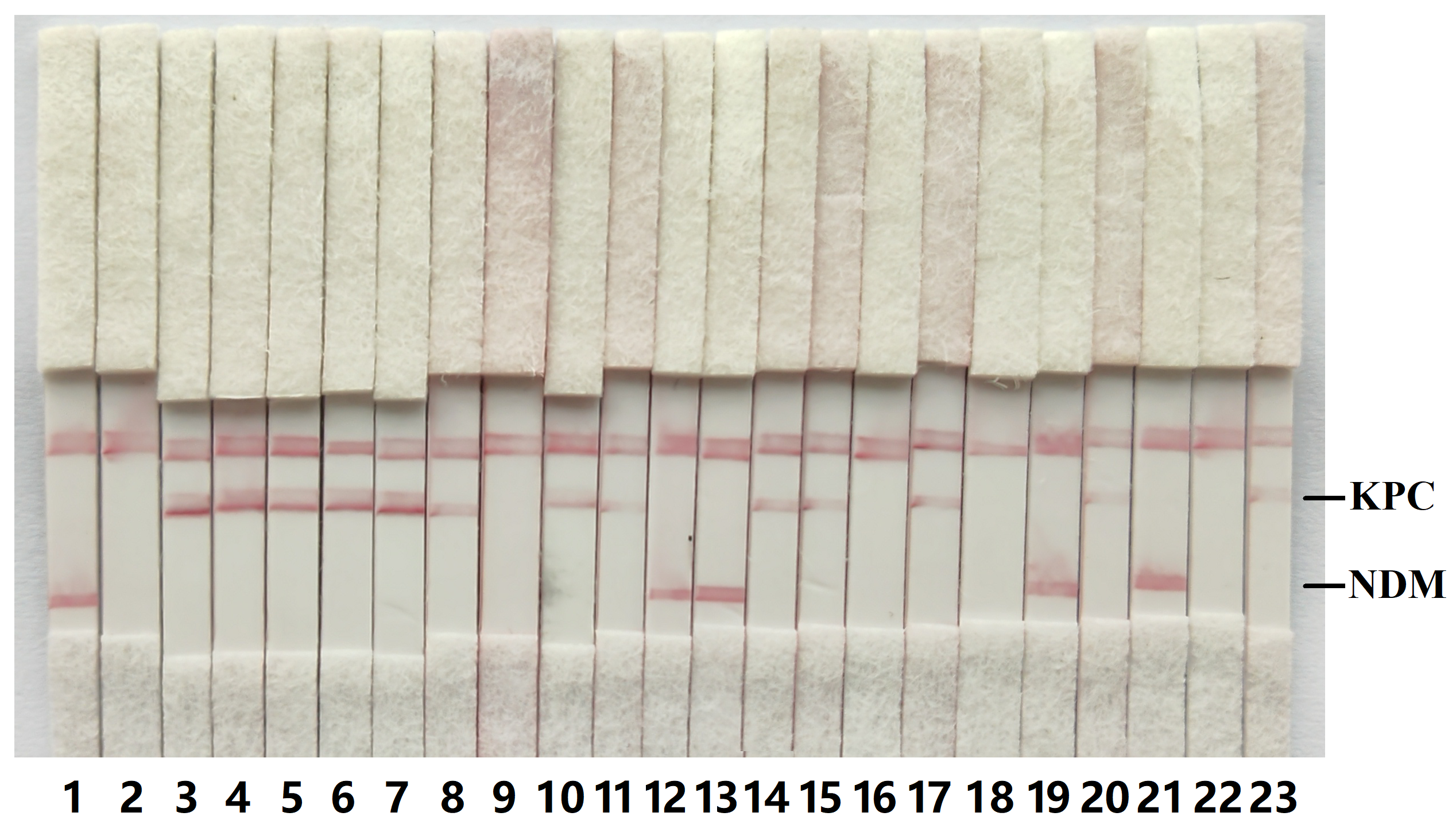
**

**Figure S3.** Results of KPC and NDM testing on 23 the Enterobacteriaceae samples using (LFT).

**Table S3** The sequencing results.

| No | **Sequences** (KPC-2) |
| --- | --- |
| 3 | GGTAACGTCATCCGCGCGGCGGTGCCGGCAGACTGGGCAGTCGGAGACAAAACCGGAACCTGCGGAGTGTATGGCACGGCAAATGACTATGAAA 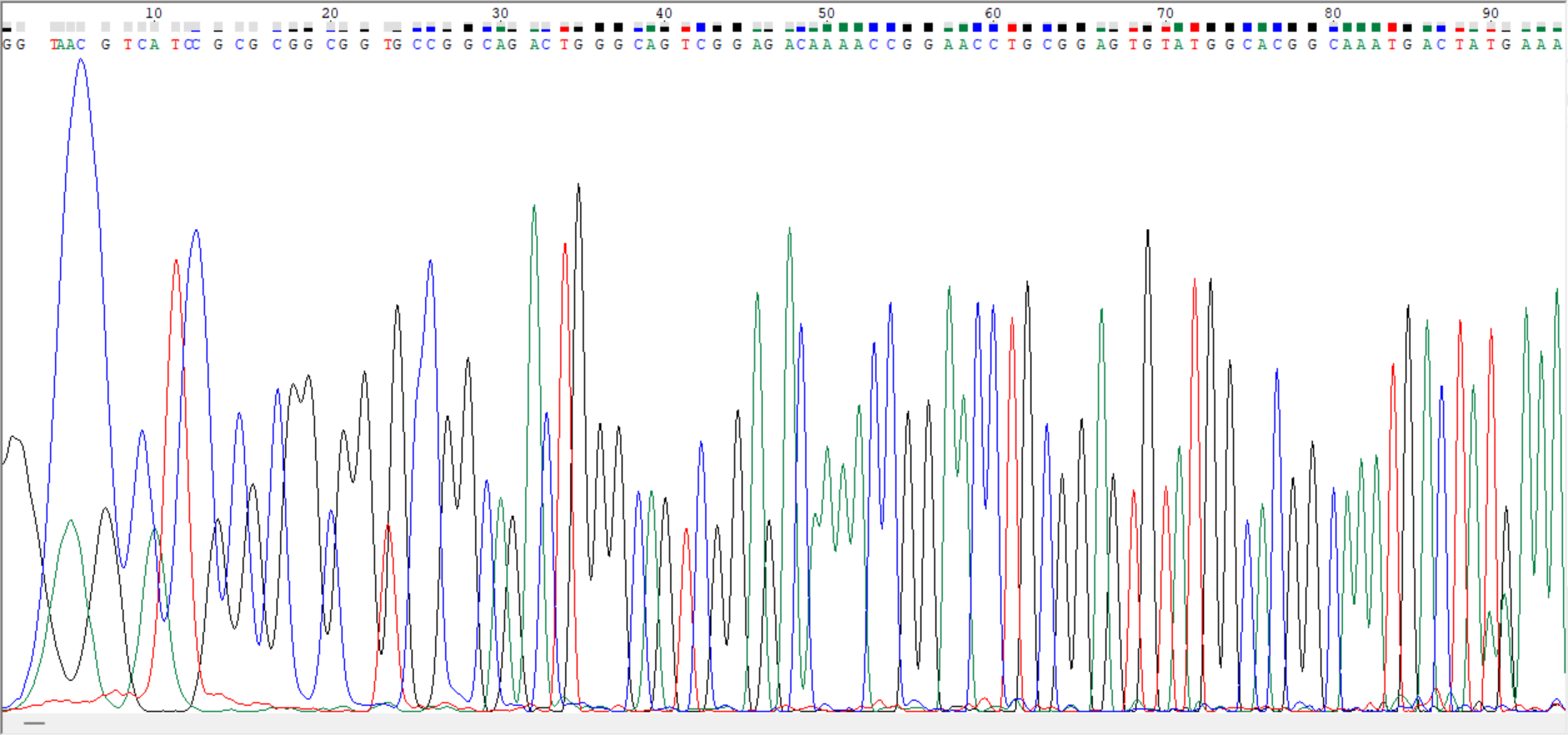 |
| 4 | GTACCGTCATCGCGCGGCGGTGCCGGCAGACTGGGCAGTCGGAGACAAAACCGGAACCTGCGGAGTGTATGGCACGGCAAATGACTAAAAAA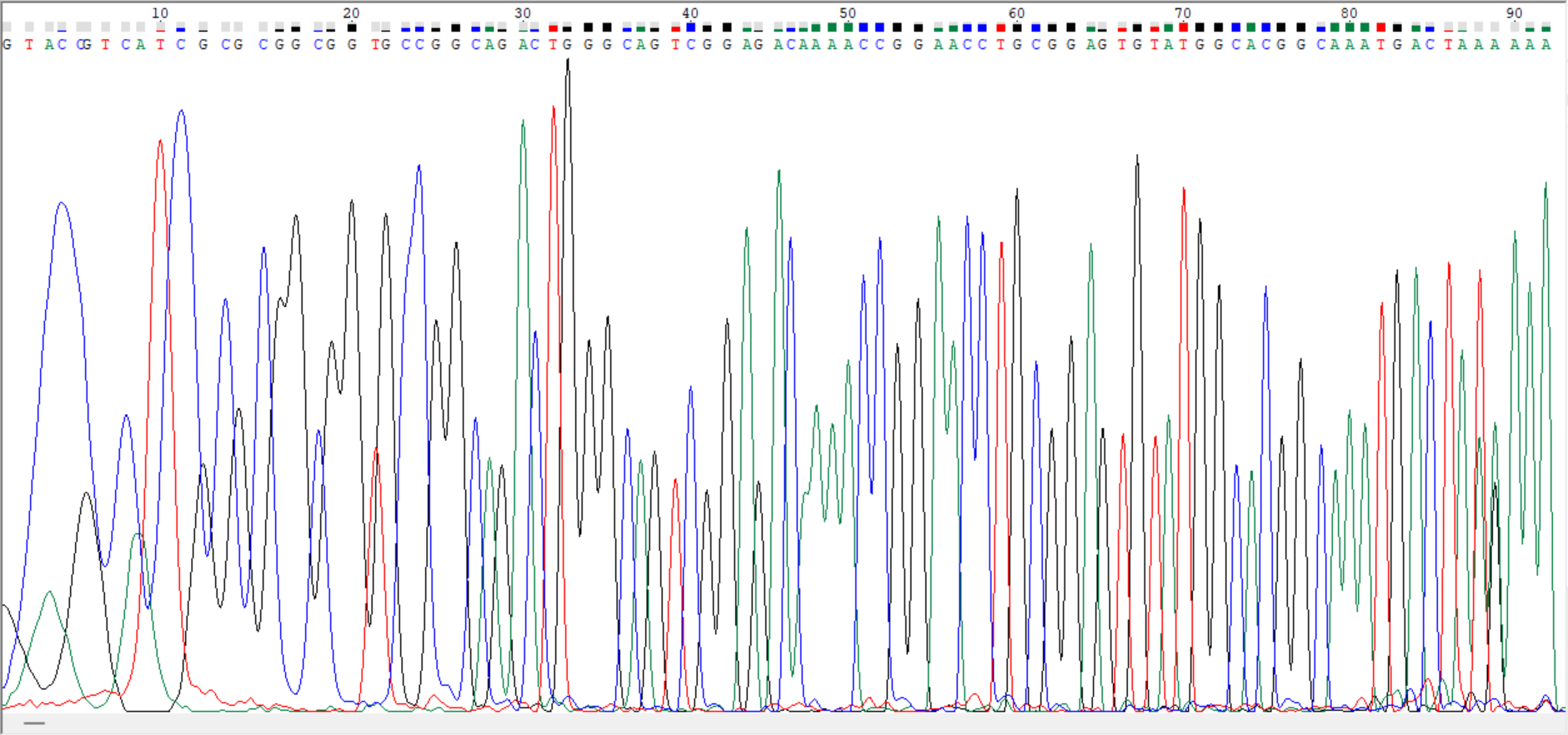 |
| 5 | CGGTCATTCCGCGCGGCGGTGCCGGCAGACTGGGCAGTCGGAGACAAAACCGGAACCTGCGGAGTGTATGGCACGGCAAATGACTATAAAA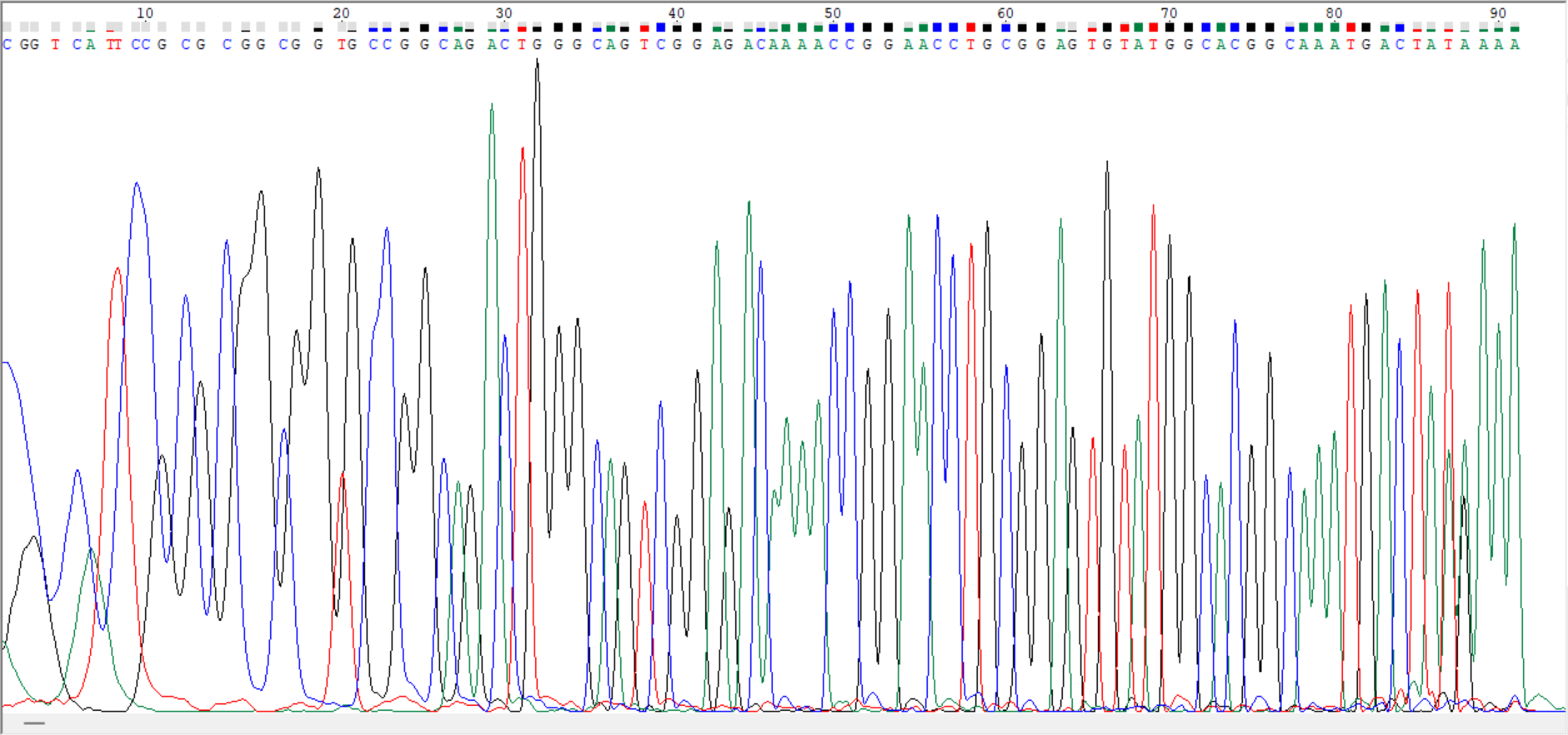 |
| 6 | GACCGGTCATCGCGCGGCGGTGCCGGCAGACTGGGCAGTCGGAGACAAAACCGGAACCTGCGGAGTGTATGGCACGGCAAATGACTATAAAA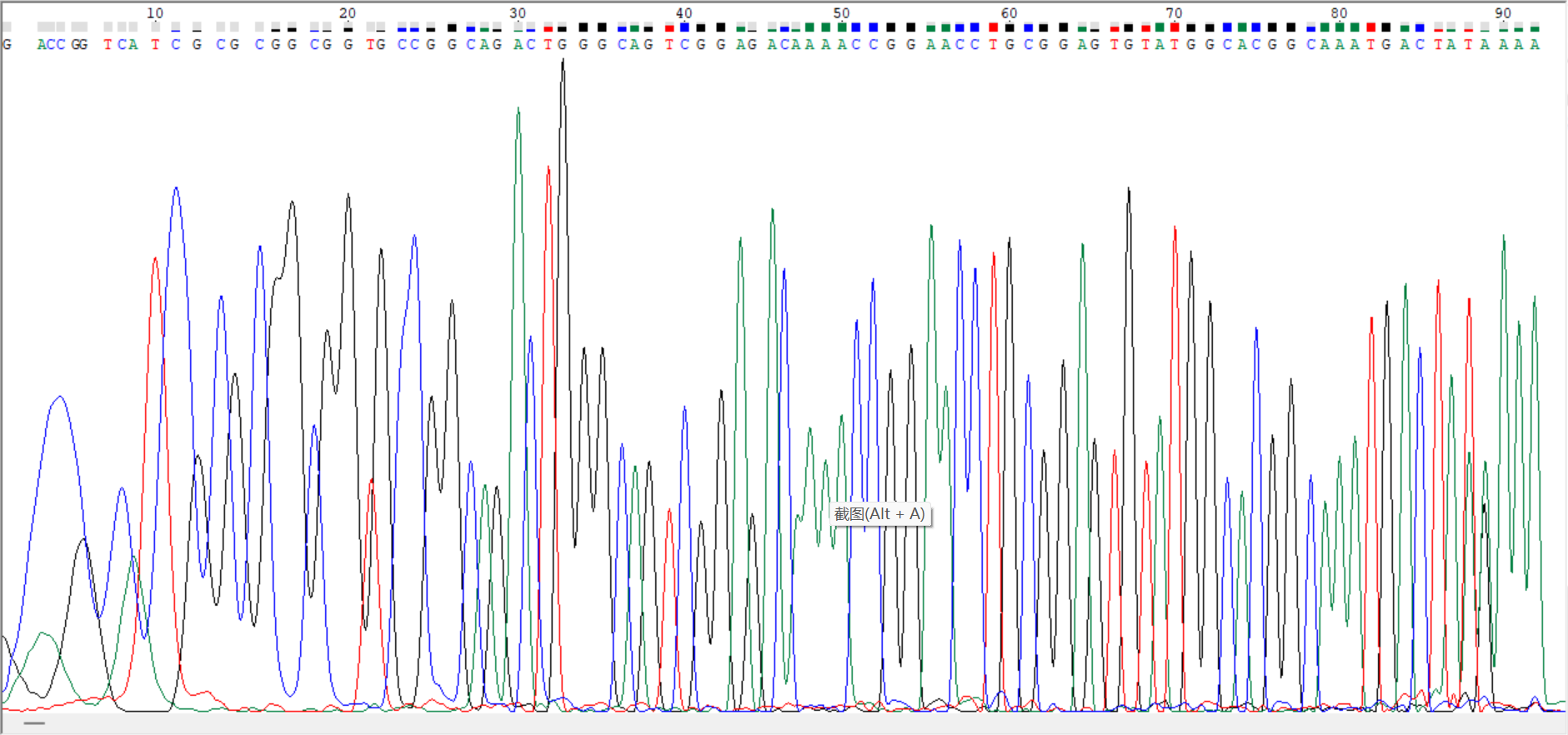 |
| 7 | CGTCCATCGCGCGGCGGTGCCGGCAGACTGGGCAGTCGGAGACAAAACCGGAACCTGCGGAGTGTATGGCACGGCAAATGACTATAAAA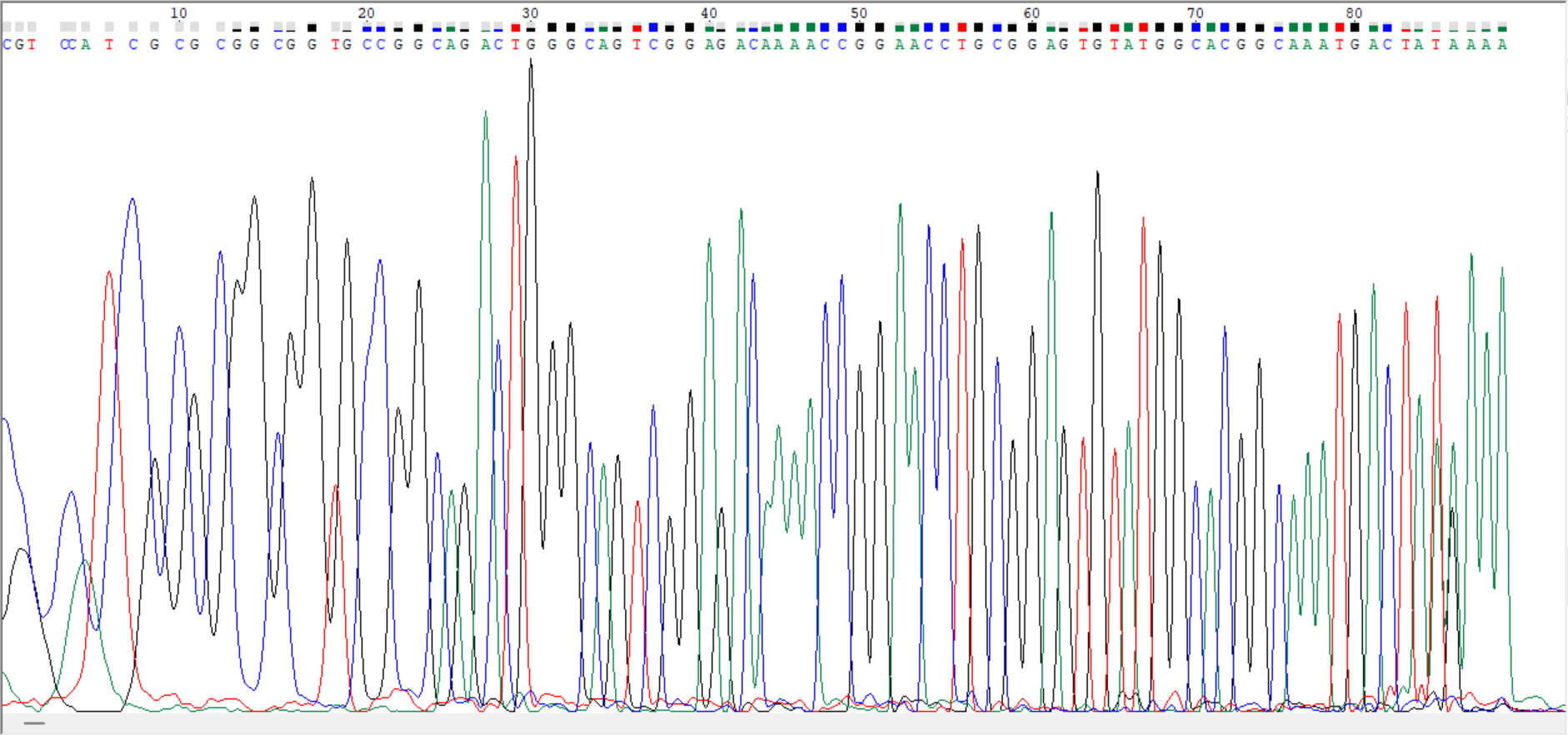 |
| 8 | GGAACCGCCATCCGCGCGGCGGTGCCGGCAGACTGGGCAGTCGGAGACAAAACCGGAACCTGCGGAGTGTATGGCACGGCAAATGACTATGAAA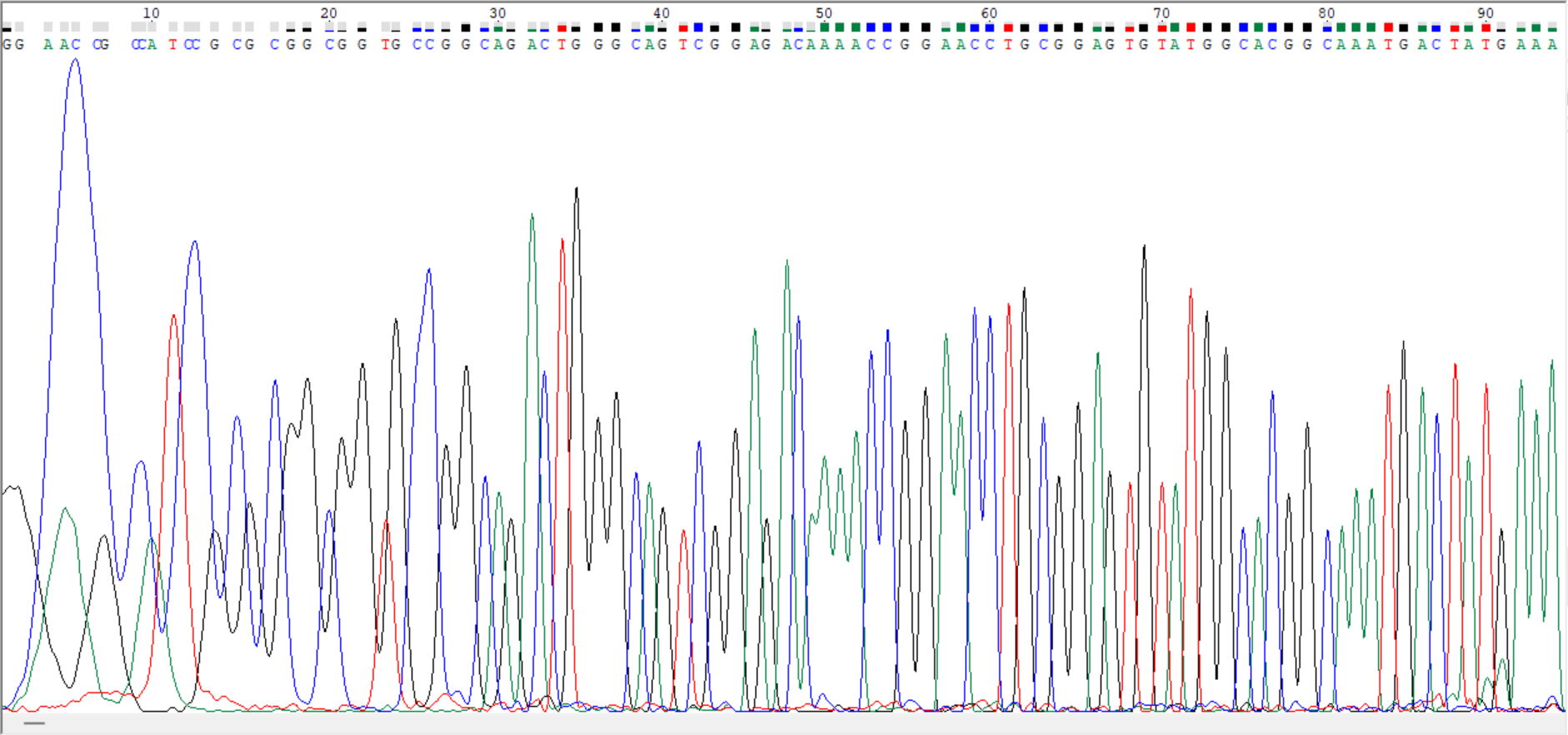 |
| 10 | GGTAACGTCATCCGCGCGGCGGTGCCGGCAGACTGGGCAGTCGGAGACAAAACCGGAACCTGCGGAGTGTATGGCACGGCAAATGACTATGAAA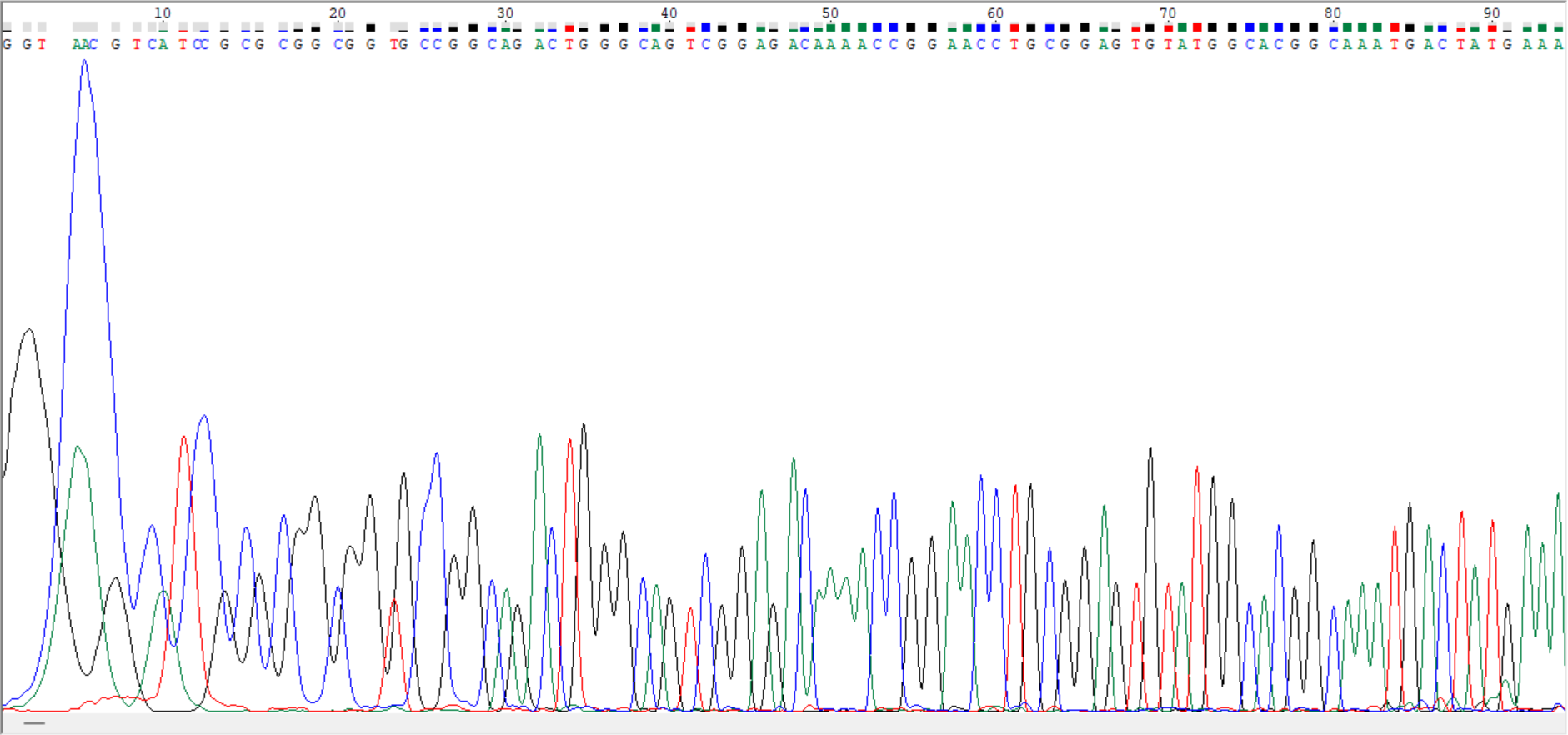 |
| 11 | GGACCGGCCATCCGCGCGGCGGTGCCGGCAGACTGGGCAGTCGGAGACAAAACCGGAACCTGCGGAGTGTATGGCACGGCAAATGACTATGAAA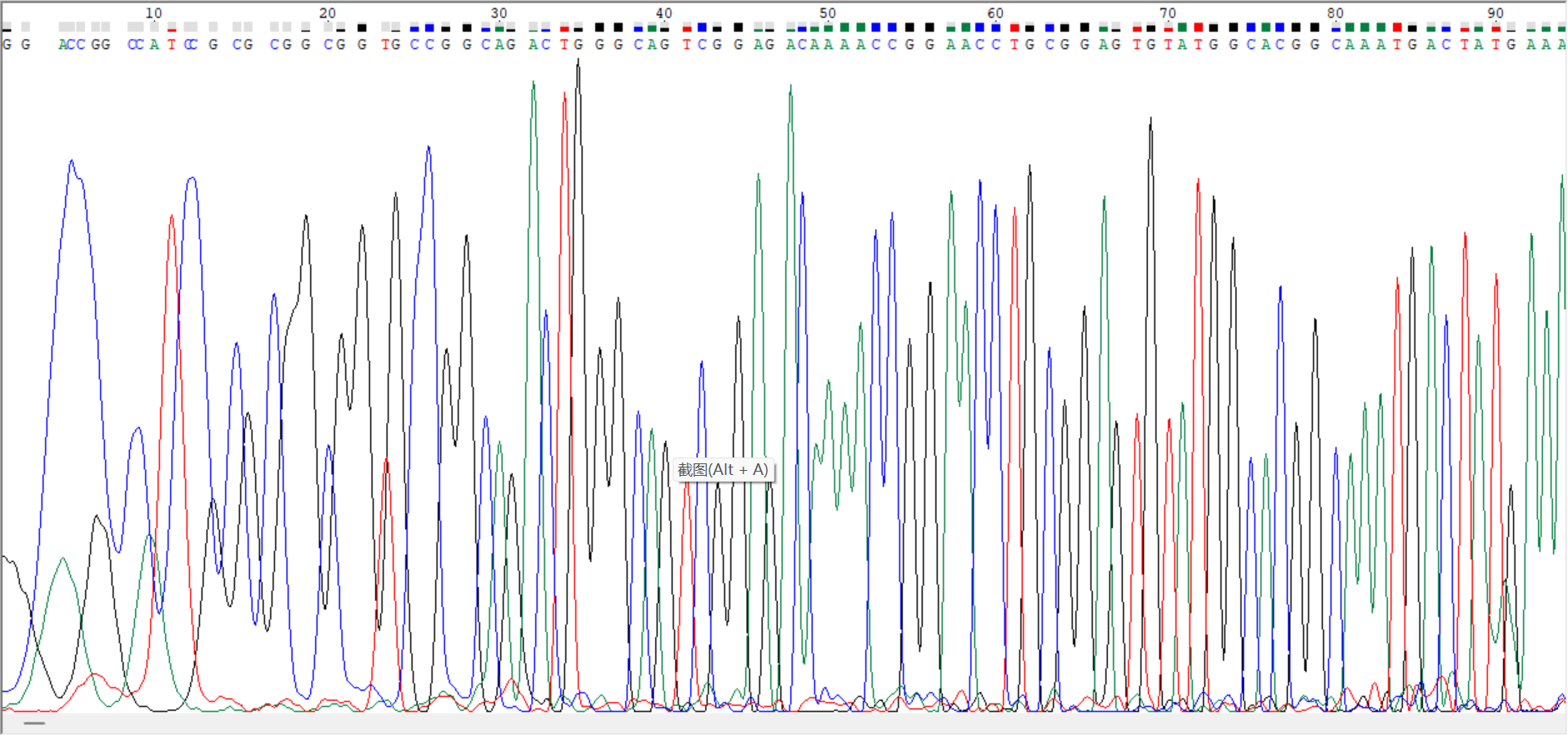 |
| 14 | GGAAACCGTCATCCGCGCGGCGGTGCCGGCAGACTGGGCAGTCGGAGACAAAACCGGAACCTGCGGAGTGTATGGCACGGCAAATGACTATGAAA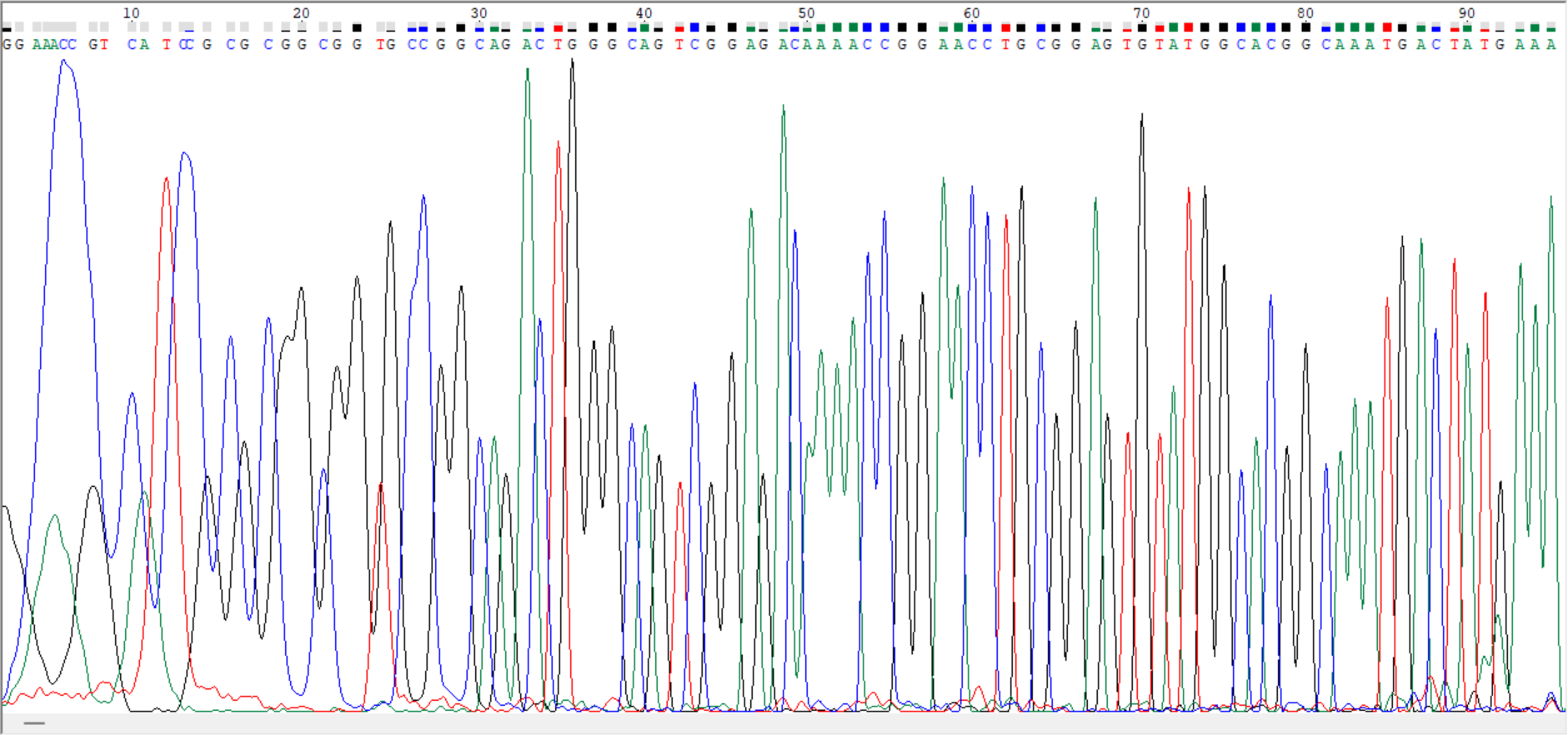 |
| 15 | GGTAAAGTCATCCGCGCGGCGGTGCCGGCAGACTGGGCAGTCGGAGACAAAACCGGAACCTGCGGAGTGTATGGCACGGCAAATGACTATGAAA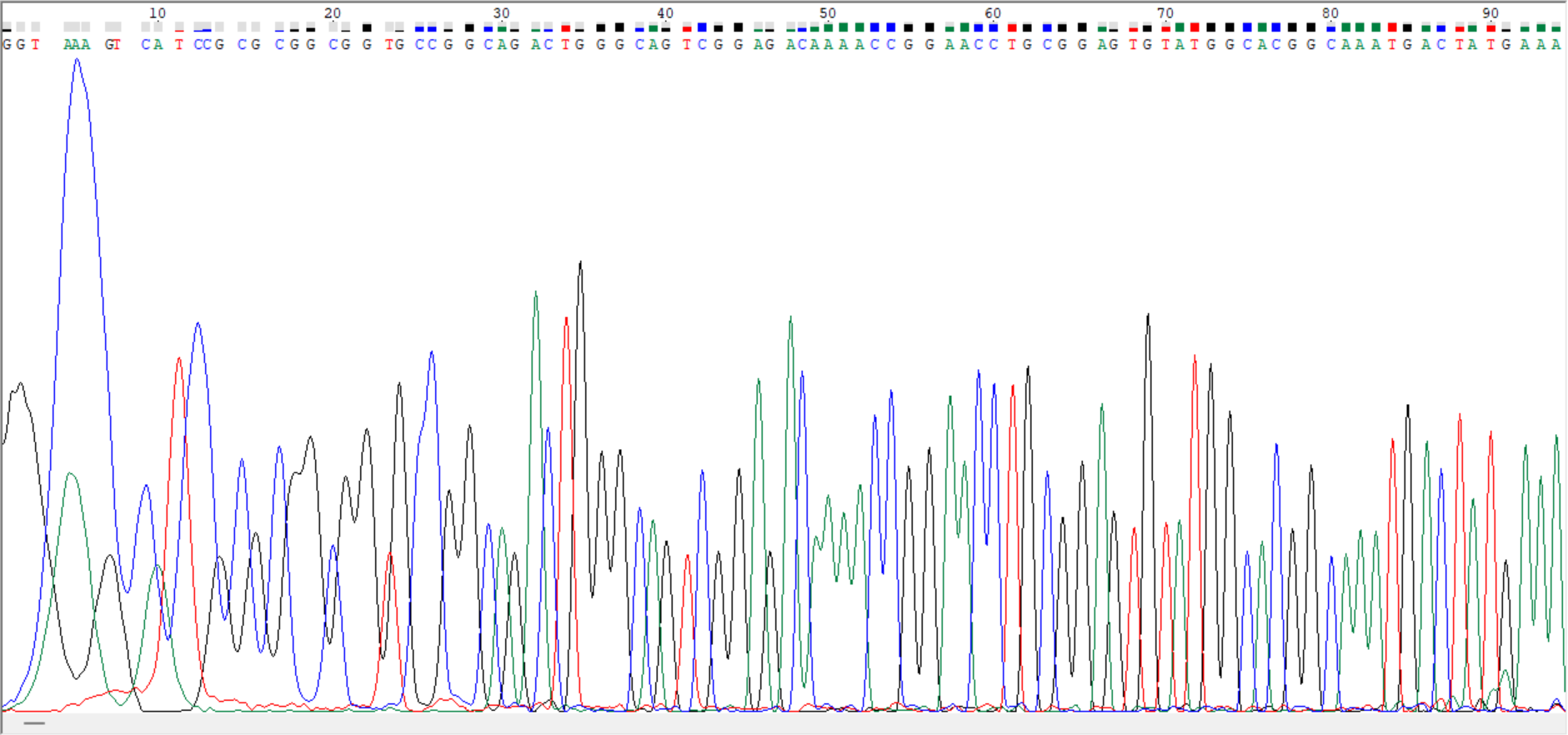 |
| 17 | TTAGTCTGCATCGCGCGGCGGTGCCGGCAGACTGGGCAGTCGGAGACAAAACCGGAACCTGCGGAGTGTATGGCACGGCAAATGACTATGAAA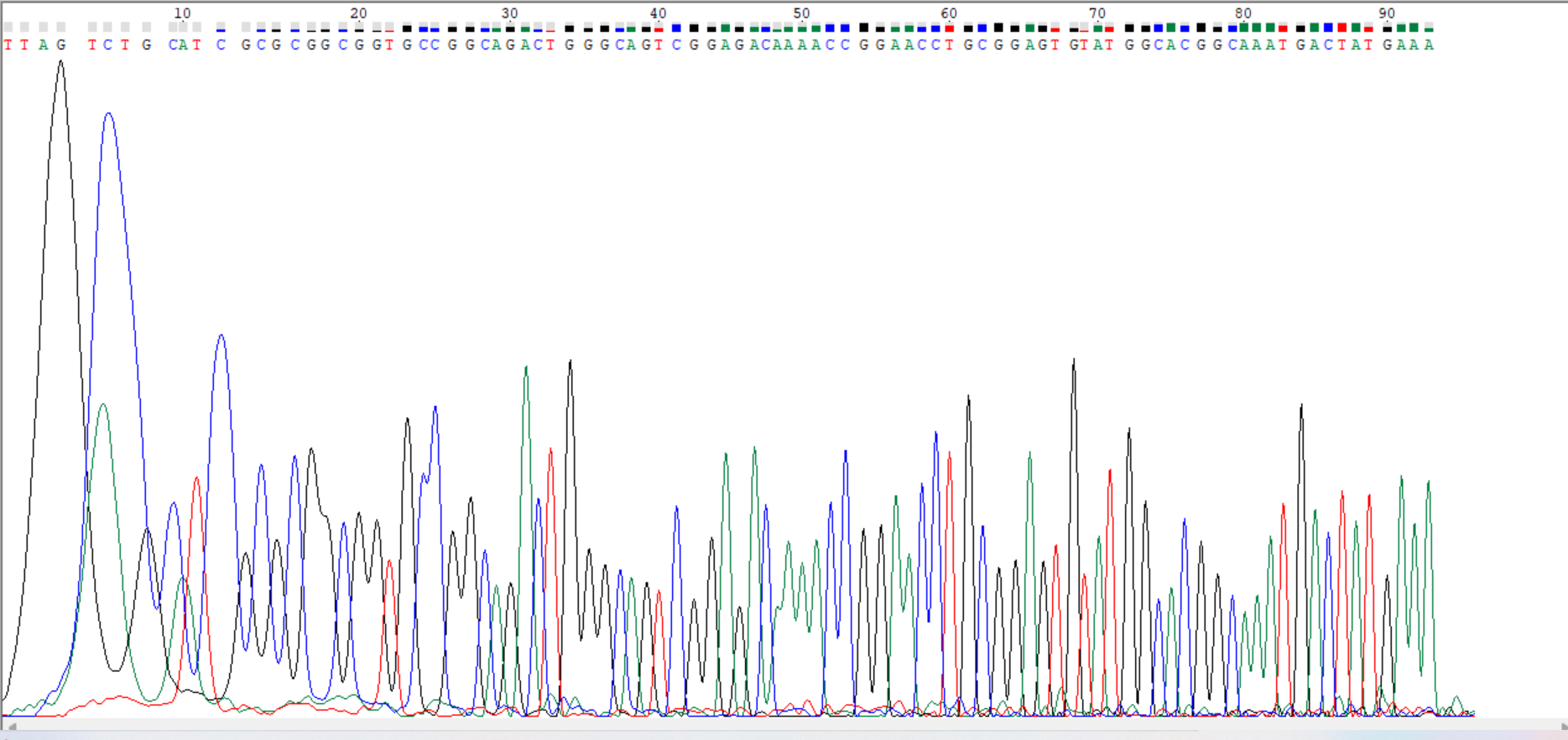 |
| 20 | TTGTATGCATCCGCGCGGCGGTGCCGGCAGACTGGGCAGTCGGAGACAAAACCGGAACCTGCGGAGTGTATGGCACGGCAAATGACTATGAAA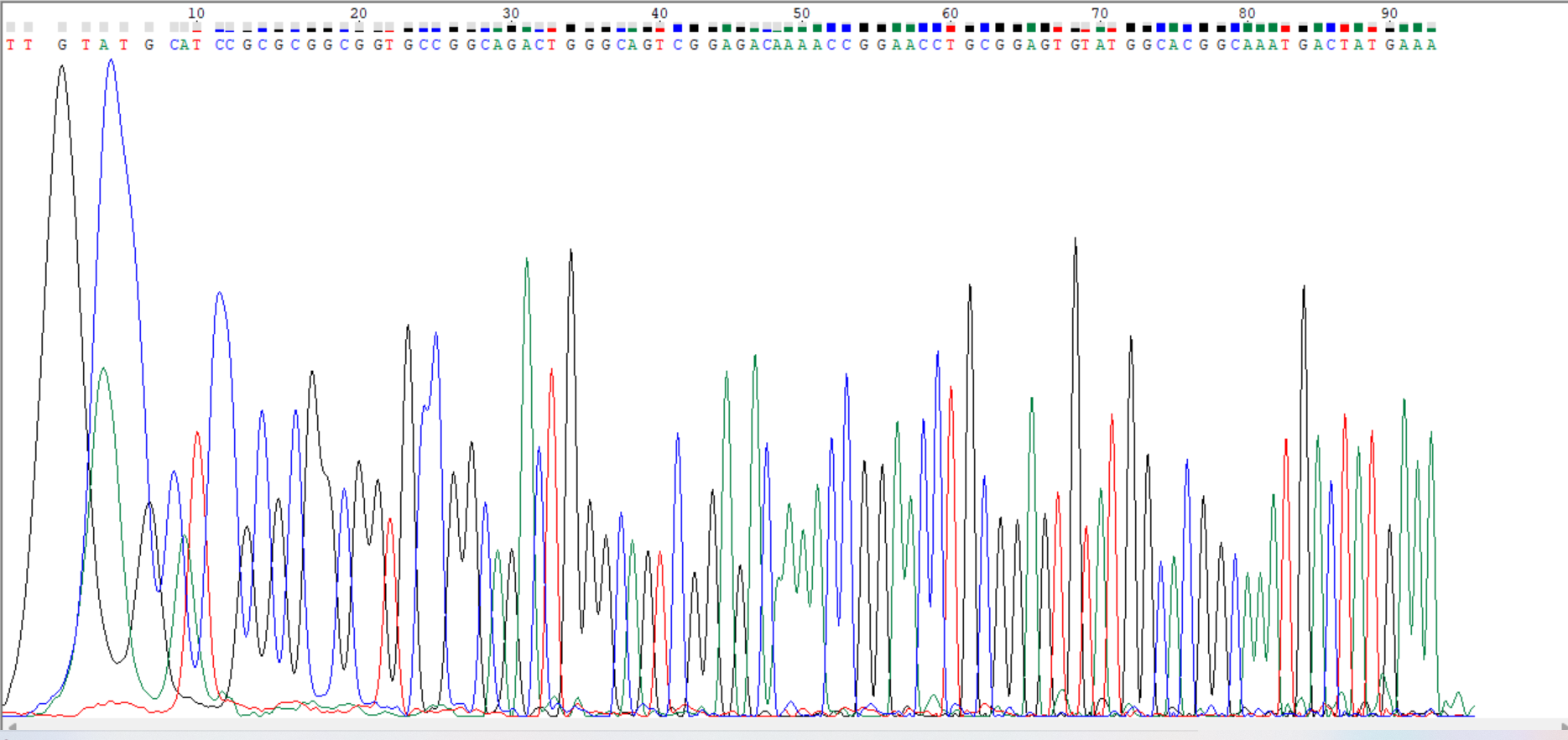 |
| 23 | TTTGCCTGCATCCGCGCGGCGGTGCCGGCAGACTGGGCAGTCGGAGACAAAACCGGAACCTGCGGAGTGTATGGCACGGCAAATGACTATGAAA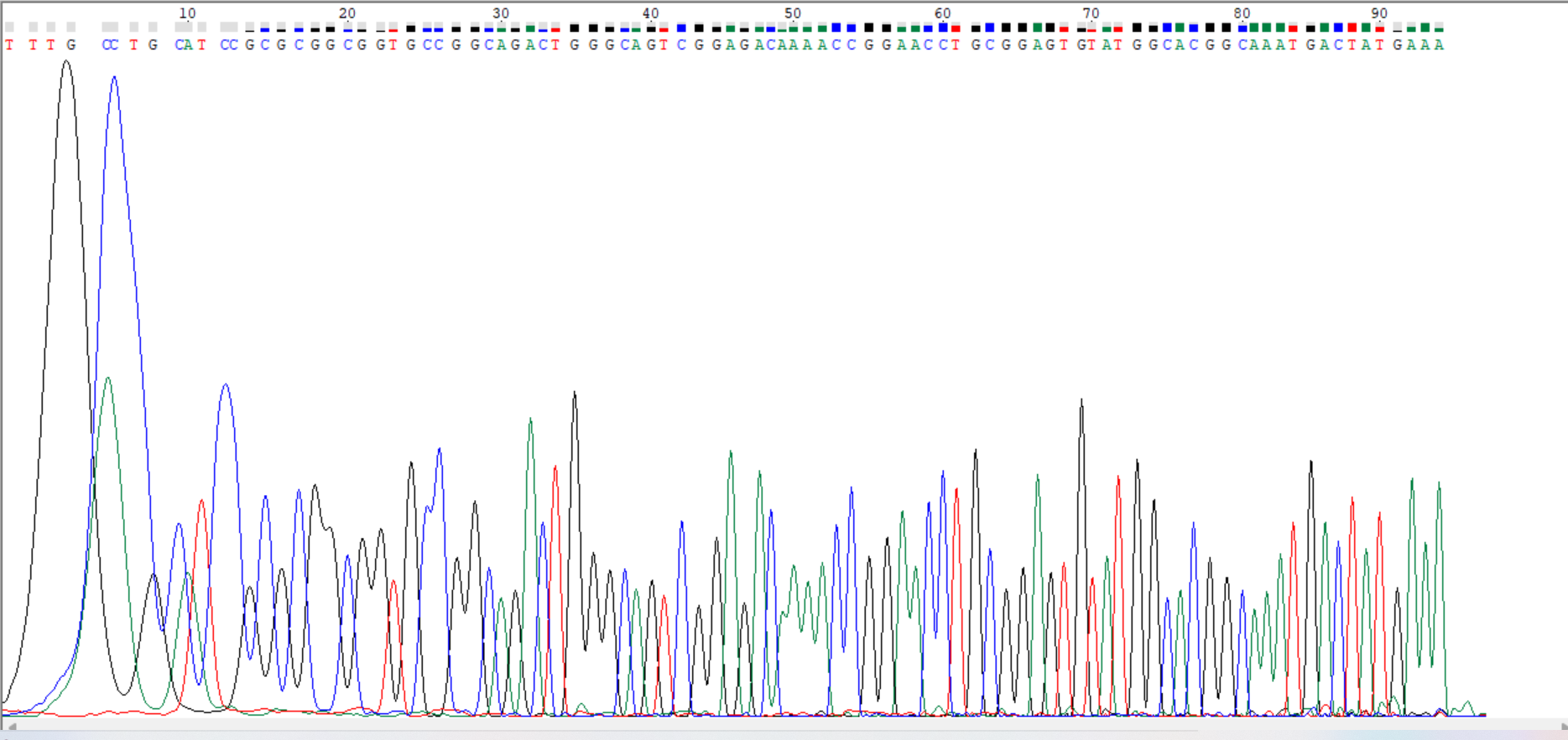 |

**Table S4** The sequencing results.

| Number | **Sequences** (NDM-1) |
| --- | --- |
| 1 | GGGGCGCGGCGTCATACCGCCCATCTTGTCCTGATGCGCGTGAGTCACCACCGCCAGCGCGACCGGCAGGTTGATCTCCTGCTTGATCCAGTTGAAAA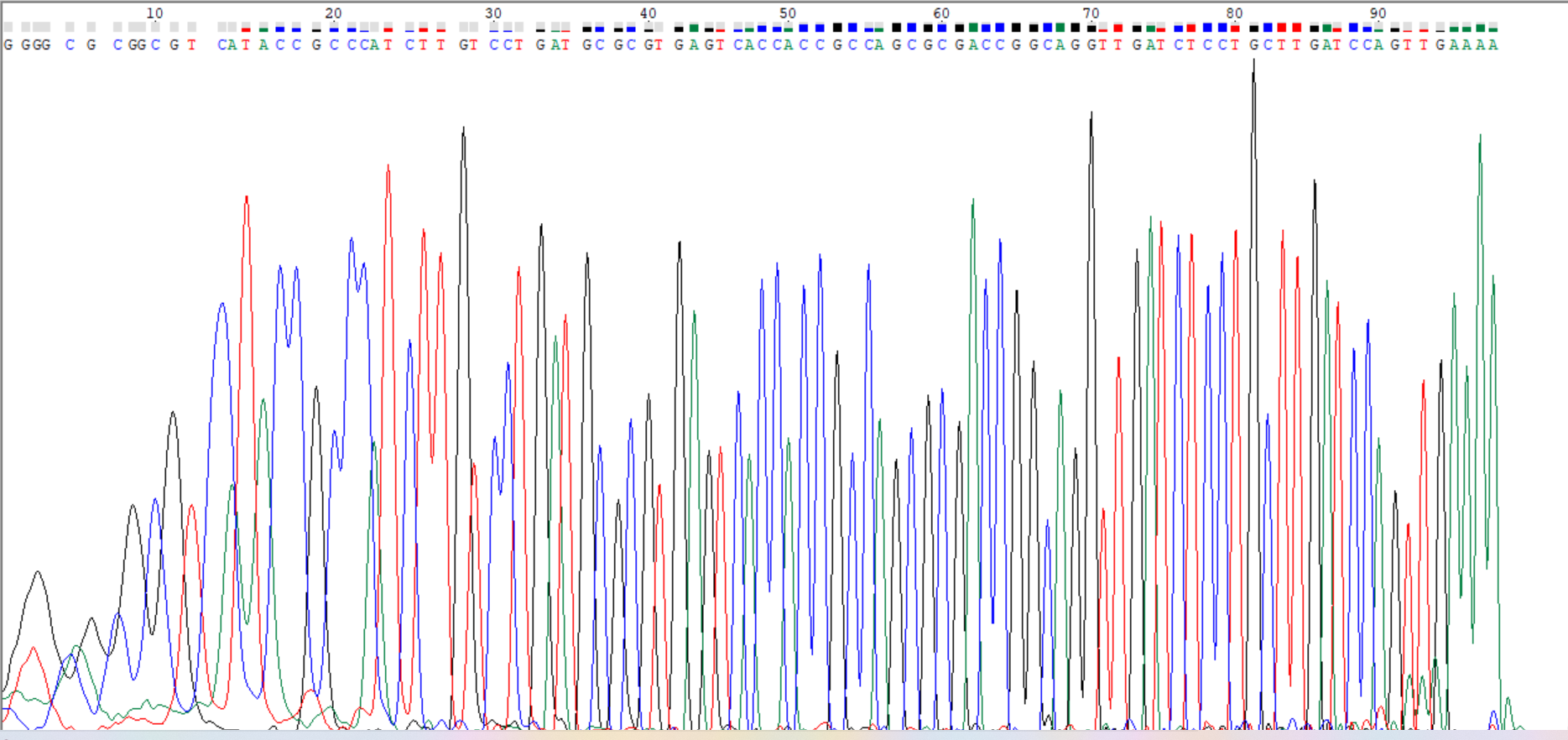 |
| 12 | GGGCGGCGCGTCCATACCGCCCATCTTGTCCTGATGCGCGTGAGTCACCACCGCCAGCGCGACCGGCAGGTTGATCTCCTGCTTGATCCAGTTGAAAA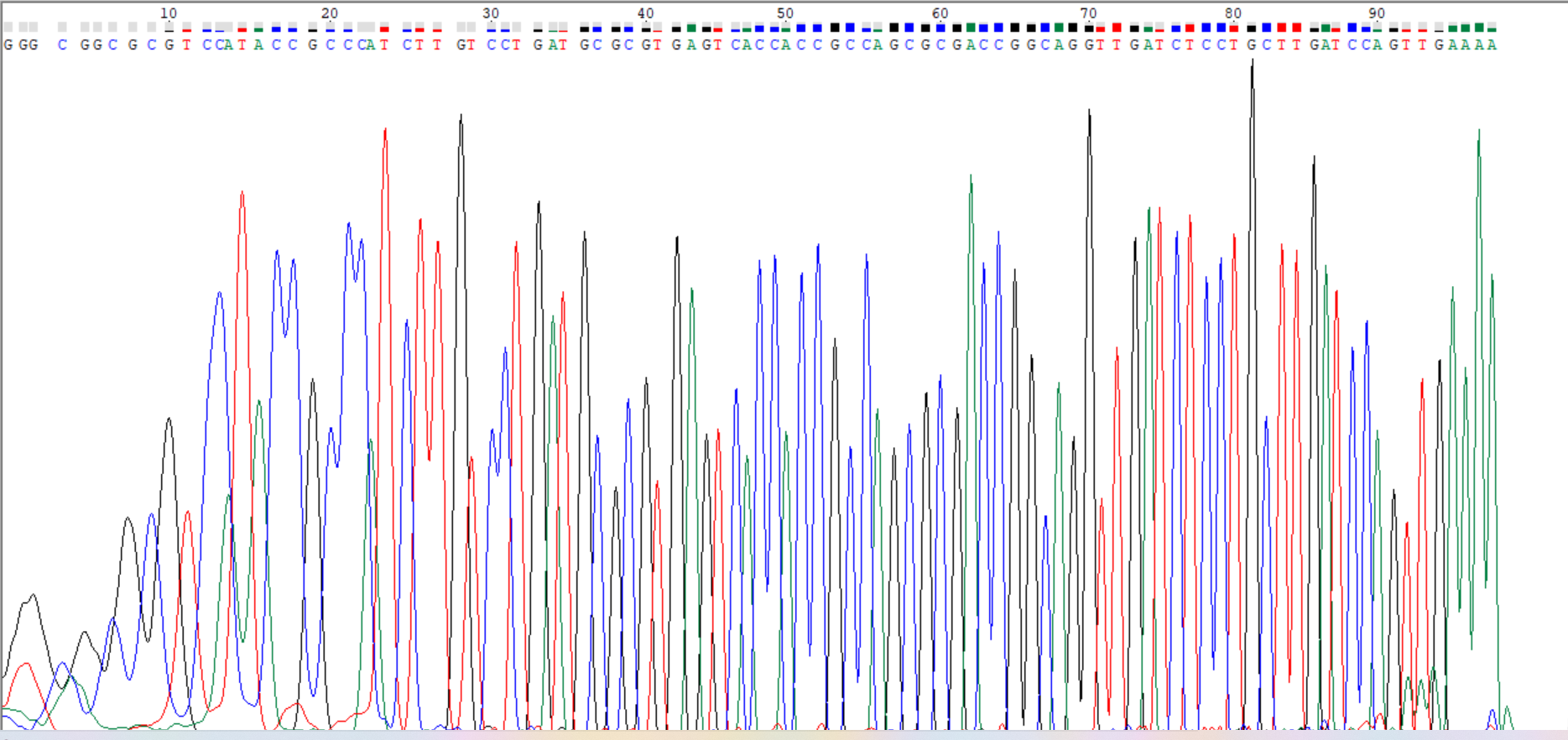 |
| 13 | CGCAGCAGCGCGTCATACCGCCCATCTTGTCCTGATGCGCGTGAGTCACCACCGCCAGCGCGACCGGCAGGTTGATCTCCTGCTTGATCCAGTTGAAAA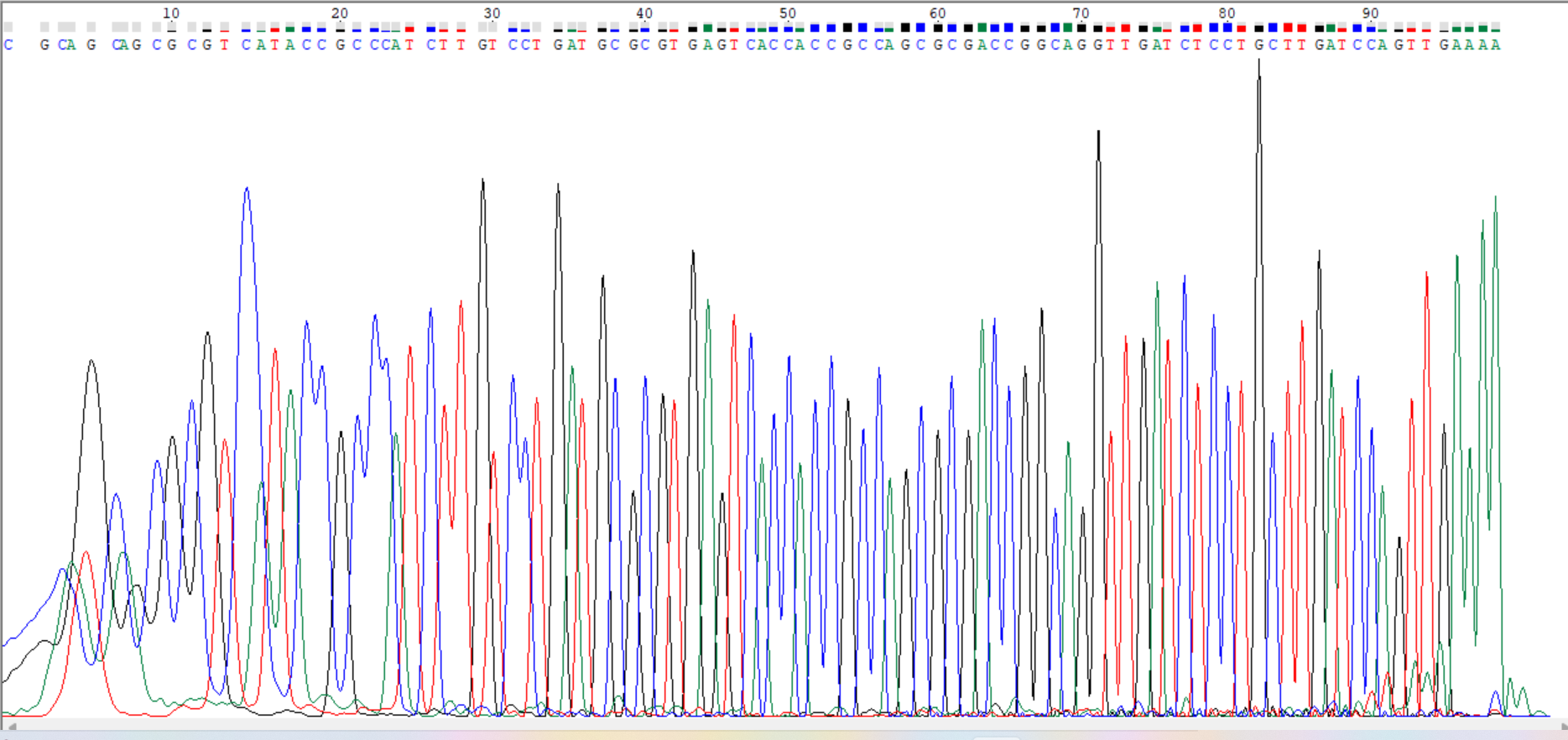 |
| 19 | ATGAGCGCGCGTCATACCGCCCATCTTGTCCTGATGCGCGTGAGTCACCACCGCCAGCGCGACCGGCAGGTTGATCTCCTGCTTGATCCAGTTGAAAA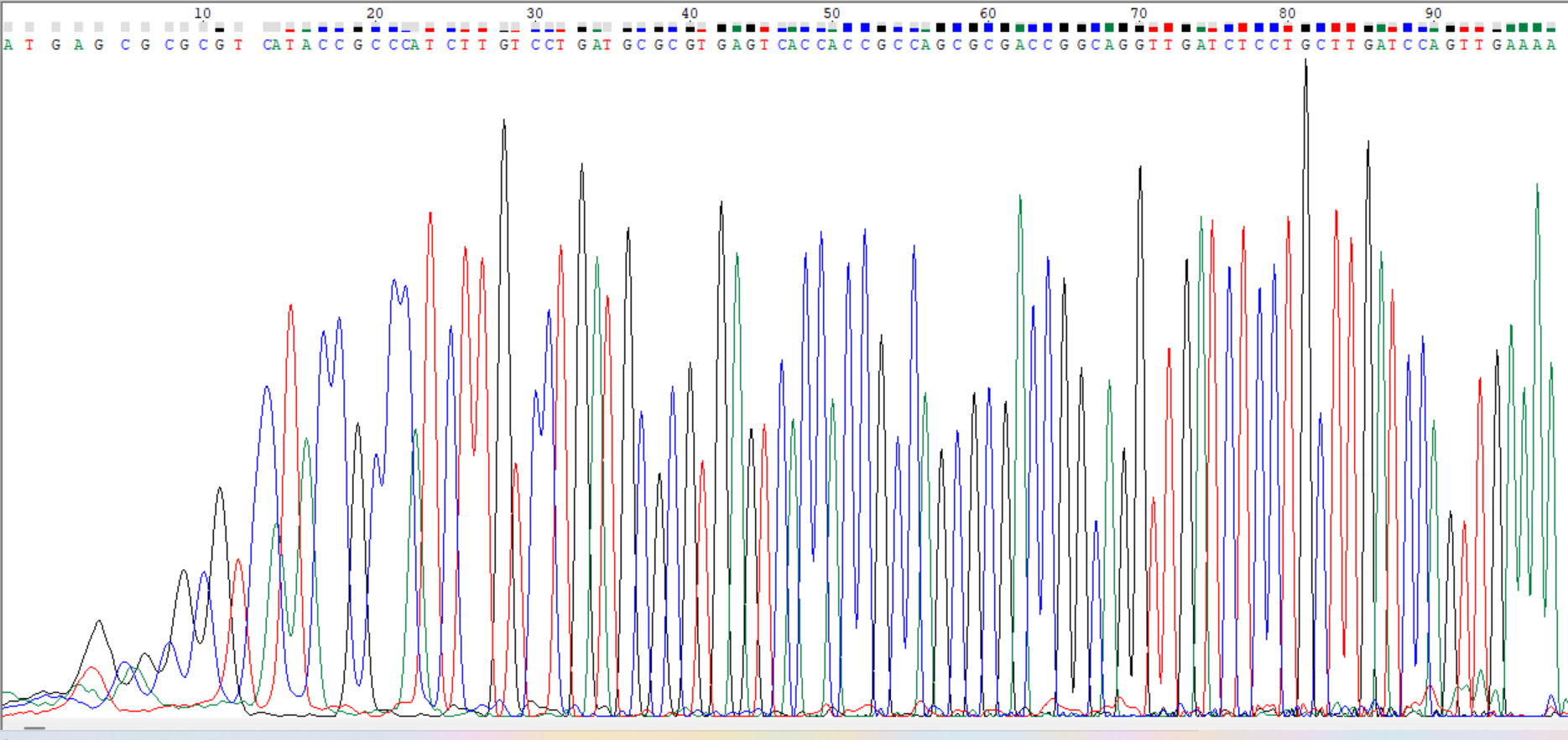 |
| 21 | CAGCAGCGCGTCATACCGCCCATCTTGTCCTGATGCGCGTGAGTCACCACCGCCAGCGCGACCGGCAGGTTGATCTCCTGCTTGATCCAGTTGAAAA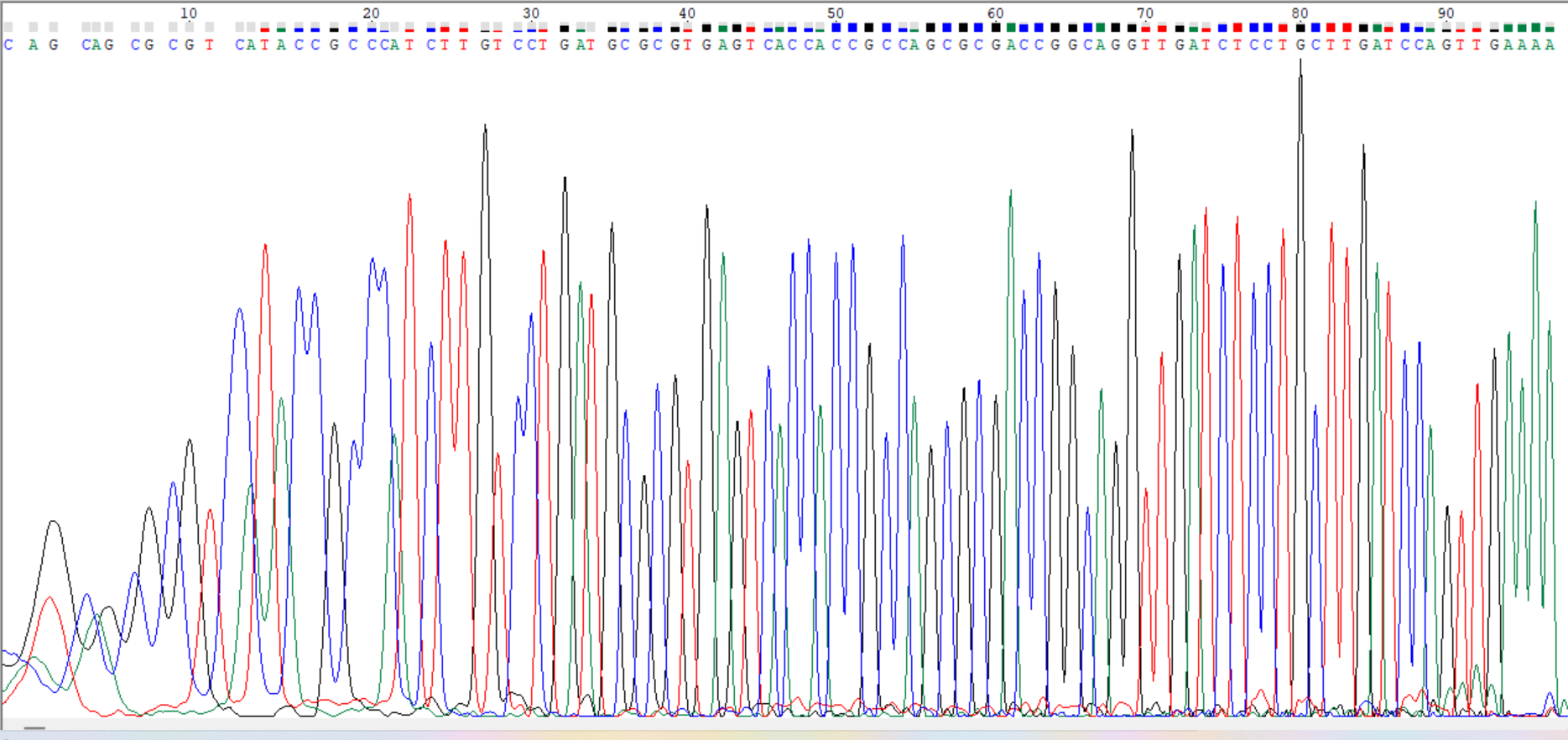 |

1. **Corresponding author.*

   *E-mail:382945020@qq.com; feiying@gmc.edu.cn*

   *^1^These authors equally contributed to this work.* [↑](#footnote-ref-2)
